# Cytokine expression in rhinovirus- vs. respiratory syncytial virus-induced first wheezing episode and its relation to clinical course

**DOI:** 10.3389/fimmu.2022.1044621

**Published:** 2022-11-14

**Authors:** Pekka Hurme, Miisa Komulainen, Marleena Tulkki, Annamari Leino, Beate Rückert, Riitta Turunen, Tytti Vuorinen, Mübeccel Akdis, Cezmi A. Akdis, Tuomas Jartti

**Affiliations:** ^1^ Department of Pediatrics and Adolescent Medicine, Turku University Hospital and University of Turku, Turku, Finland; ^2^ Swiss Institute of Allergy and Asthma Research (SIAF), University of Zürich, Christine Kühne-Center for Allergy Research and Education (CK-CARE), Davos, Switzerland; ^3^ New Children’s Hospital, Helsinki University Hospital and University of Helsinki, Helsinki, Finland; ^4^ Institute of Biomedicine, University of Turku, Turku, Finland; ^5^ Department of Clinical Microbiology, Turku University Hospital, Turku, Finland; ^6^ PEDEGO Research Unit, Medical Research Center, University of Oulu, Oulu, Finland; ^7^ Department of Pediatrics and Adolescent Medicine, Oulu University Hospital, Oulu, Finland

**Keywords:** bronchiolitis, cytokine, respiratory syncytial virus, rhinovirus, wheezing

## Abstract

Rhinovirus (RV) and respiratory syncytial virus (RSV) are common causes of bronchiolitis. Unlike an RSV etiology, an RV etiology is associated with a markedly increased risk of asthma. We investigated the cytokine profiles of RV- and RSV-induced first wheezing episode and their correlation with prognosis. We recruited 52 sole RV- and 11 sole RSV-affected children with a severe first wheezing episode. Peripheral blood mononuclear cells (PBMCs) were isolated during acute illness and 2 weeks later and stimulated *in vitro* with anti-CD3/anti-CD28. Culture medium samples were analyzed for 56 different cytokines by multiplex ELISA. Recurrences were prospectively followed for 4 years. In adjusted analyses, the cytokine response from PBMCs in the RV group was characterized by decreased expression of interleukin 1 receptor antagonist (IL-1RA), interleukin 1 beta (IL-1β), and monocyte chemoattractant protein-1 (MCP-1) and increased expression of eosinophil chemotactic protein 2 (eotaxin-2), thymus- and activation-regulated chemokine (TARC), and epithelial-derived neutrophil-activating peptide 78 (ENA-78) in the acute phase and increased expression of fractalkine in the convalescent phase compared to those in the RSV group. An analysis of the change in cytokine expression between study points revealed an increased expression of fractalkine and IL-1β and decreased expression of I-309 (CCL1) and TARC in the RV group compared to those in the RSV group.. Considering hospitalization time, a significant non-adjusted group × cytokine interaction was observed in the levels of interferon gamma (IFN-γ), macrophage-derived chemokine (MDC), IL-1RA, and vascular endothelial growth factor (VEGF), indicating that a higher expression of cytokine was associated with shorter hospitalization time in the RSV group but not in the RV group. A significant interaction was also found in interleukin 6 (IL-6), but the cytokine response was not associated with hospitalization time in the RSV or RV group. In the RV group, increased expression of I-309 (CCL1) and TARC was associated with fewer relapses within 2 months, and decreased expression of interleukin 13 (IL-13) and increased expression of I-309 (CCL1) were associated with less relapses within 12 months. Differences in cytokine response from PBMCs were observed between RV- and RSV-induced first severe wheezing episode. Our findings also reveal new biomarkers for short- and medium-term prognosis in first-time wheezing children infected with RV or RSV.

## Introduction

Up to a third of all children suffer from bronchiolitis during the first 2 years of life, and it is the most common cause for hospitalization in children. Respiratory syncytial virus (RSV) and rhinovirus (RV) are the most common etiologic agents (1). RSV is most commonly found in children under 12 months of age, but RV starts to dominate thereafter (1, 2). The “common” bronchiolitis diagnosis has been criticized as too obscure, and more specific classification according to virus entities has been anticipated (3).

Both RSV and RV target and replicate in epithelial cells of the airways that result in innate immune activation and a rapid burst of type I/III interferons (IFNs) (1). This is followed by the induction of several cytokines and chemokines, leading to epithelial cell apoptosis, necrosis, epithelial sloughing, and mucus overproduction. Typically, cytopathic effects are more severe in RSV infection. In contrast to RSV, atopic predisposition and a distinct single polymorphism in the *CDHR3* gene or 17q locus increase the risk for more severe RV-induced illness and a more compromised long-term prognosis (4–6). Studies in human and murine models have shown that RV infections of airway epithelial cells are inducers of type 2 innate cytokines, such as interleukin (IL)-25 and IL-33, which subsequently initiate or boost type 2 immunity in the lungs *via* IL-5- and IL-13-producing group 2 innate lymphoid cells (ILC2) and T helper 2 (Th2) cells (7–9).

Although RSV typically causes more severe bronchiolitis, an RV etiology is associated with a higher risk of recurrent wheezing and asthma than does an RSV etiology (1, 2, 6, 10–12). The exact mechanism for these differences is not known, and there are no precise data about the immunopathologic differences between the two major etiologic agents of bronchiolitis, RSV and RV. Therefore, we aimed to investigate the cytokine profiles of RV- vs. RSV-induced first severe wheezing episode and their relation to short- and long-term outcomes. We hypothesized that potential differences in the cytokine response from peripheral blood mononuclear cells (PBMCs) of children with virus-induced acute wheezing due to RV compared to RSV may be linked to prognosis.

## Materials and methods

### Subjects

**Figure 1:**
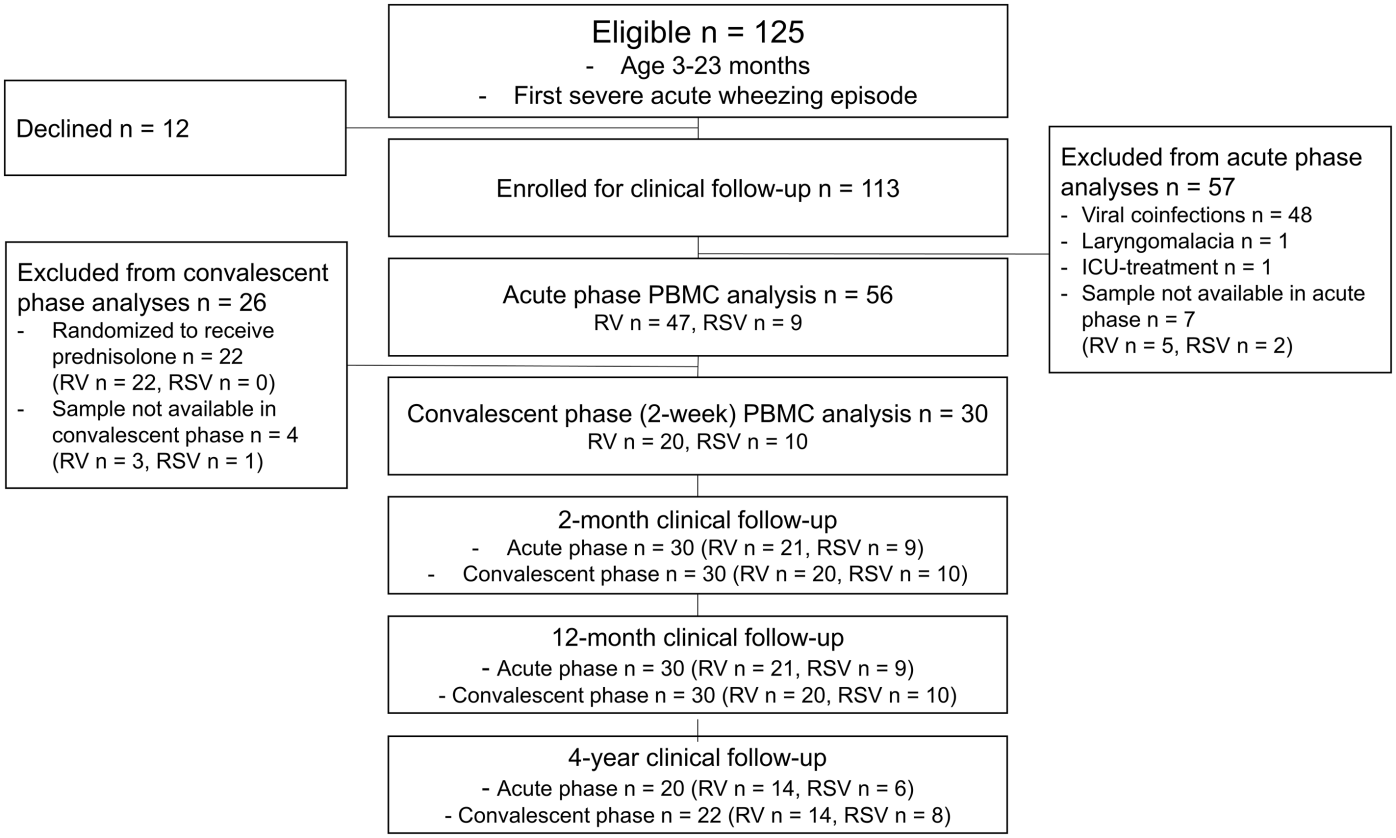
Study flowchart. Patients with cytology data were included. ICU, intensive care unit; PBMC, peripheral blood mononuclear cell; RSV, respiratory syncytial virus; RV, rhinovirus.

The study population was part of the Vinku2 study in which RV-affected first-time wheezing children were randomized to receive oral prednisolone (2 mg/kg/day for 3 days) or placebo (updated version for 7-year follow-up NCT00731575, original version EudraCT 2006-007100-42) (13). Its recruitment was carried out in 2007–2010 in the Department of Pediatrics, Turku University Hospital (Turku, Finland). The main inclusion criteria for the current analysis were age 3–23 months, delivery at ≥36 gestational weeks, first wheezy episode (parental report and confirmed from medical records), sole steroid-naive RV or RSV infection detected in a nasopharyngeal aspirate sample by polymerase chain reaction (PCR), and written informed consent from a parent or guardian. The exact PCR procedure has been previously described in detail (14). The main exclusion criteria consisted of chronic non-atopic illness, previous systemic or inhaled corticosteroid treatment, or the need for intensive care unit treatment (**Figure 1**). The study was approved by the Ethics Committee of Turku University Hospital and commenced only after obtaining written informed consent from the guardians.

### Study protocol

The need for hospitalization was decided by an on-duty study physician independent of the study. Recruitment to the study was done by the study physician. At study entry, the guardian filled in a standard questionnaire on host and environmental risk factors for asthma. The child was then physically examined by the study physician, a nasopharyngeal aspirate sample was obtained for viral diagnostics using a standardized procedure (15), and a baseline blood sample was drawn. The children were randomized to be given either oral prednisolone or a placebo after a positive RV PCR test (prednisolone receivers were excluded from subsequent analyses). The second blood sample was drawn at the convalescent phase, 2 weeks after study entry. The Vinku2 study used follow-up protocols, including daily symptom diaries, for the first 2 months. Thereafter, new breathing difficulties were prospectively recorded in a diary, and follow-up visits at 2 weeks, 2 months, 12 months, and 4 years were scheduled by the study physicians. Furthermore, the guardian was asked to bring the child to the study physician each time the child had breathing difficulties.

### Study aims

We had several aims in the study:

1. To compare the cytokine response from anti-CD3/anti-CD28-stimulated PBMCs between RV- and RSV-affected severe first-time wheezing children (80% hospitalized and 20% treated in the emergency room of a tertiary hospital) in the acute phase and convalescent phase 2 weeks later.
2. To compare the cytokine expression between RV species, that is, *A*, *B*, and *C* species.
3. To investigate whether the cytokine expression is associated with the virus genome load (RV).
4. To assess whether the cytokine expression is associated with recurrences (i.e., new physician-confirmed wheezing episodes within the subsequent 2 months and 12 months) and asthma at 4 years.

### Definitions

Asthma refers to recurring airway obstruction and intermittent symptoms of increased airway responsiveness to triggering factors, such as exercise, allergen exposure, and viral infection. Wheezing refers to expiratory breathing difficulty with bilateral high-pitched sounds during expiration. Wheezing episodes accompanied by sole RV or RSV detection by PCR were called RV- or RSV-induced wheezing episodes, respectively. Atopy was defined as a positive immunoglobulin (Ig) E antibody (≥.35 kU/L) to any of the following allergens: codfish, cow’s milk, egg, peanut, soybean, wheat, cat, dog, horse, birch, mugwort, timothy, *Cladosporium herbarum*, and *Dermatophagoides pteronyssinus* (Phadiatop Combi^®^, Phadia, Uppsala, Sweden). Aeroallergen sensitization was defined as a positive IgE antibody to any of the latter eight allergens. Perennial aeroallergen sensitization was defined as positive IgE antibodies to dog, cat, or *D. pteronyssinus*. Birch, mugwort, timothy, and *C. herbarum* were considered seasonal aeroallergens. Eczema was defined as a physician diagnosis according to typical symptoms that included pruritus, typical morphology, and chronicity of the disease. Eczema was defined as atopic eczema if a child had atopy (defined above). Type 1 immunity refers to the activity of T helper 1 (Th1) cells, group 1 innate lymphoid cells (ILC1), neutrophils, and classically activated macrophages. Type 2 immunity refers to the activity of Th2 cells, ILC2, eosinophils, mast cells, basophils, and IL-4- and IL-13-activated macrophages.

### Virus detection

Nasal swabs (nylon flocked dry swab, 520CS01, Copan, Brescia, Italy) were dipped into the nasopharyngeal aspirate and stored at -70°C until analyzed. Virus analyses were performed as described previously (16, 17). Briefly, the swab was diluted in 1 ml phosphate-buffered saline, and respiratory viruses and RV genome copy numbers were analyzed from extracted nucleic acids. RV A, B, and C, enteroviruses, and RSV A and B were detected by RT-PCR using in-house reverse transcriptase PCR at the Virus Diagnostic Laboratory, Department of Virology, University of Turku (18, 19). A multiplex PCR test (Seeplex RV12 ACE Detection, Seegene, Seoul, Korea) was used for detection of RV A and B, RSV A and B, parainfluenza virus types 1–3, human metapneumovirus, adenovirus, coronavirus (229E, NL63, OC43, and HKU1), and influenza A and B viruses. Human bocavirus-1 was analyzed using PCR and serology, as previously described (20). The blood eosinophil count (B-Eos) and serum levels of allergen-specific IgE were analyzed using routine diagnostics of the Central Laboratory of Turku University Hospital. Serum 25-hydroxyvitamin D measurements were performed by liquid chromatography-tandem mass spectrometry at Massachusetts General Hospital (Boston, MA, USA).

### Peripheral blood mononuclear cell processing (isolation, cell cultures, and stimulation) and cytokine analyses

Blood samples for PBMCs were collected during the acute illness and convalescent phase (2-week follow-up). On each time point, the collected blood was stored on a rocking shaker in room temperature, and PBMC isolation of the samples was performed on the same day. PBMCs were separated from the blood using Ficoll-Paque™ PLUS (GE Healthcare, Amersham, UK) density gradient centrifugation according to the manufacturer’s protocol. Further processing of the samples was done within the same day. PBMCs (>95% live cells) were then stimulated with anti-CD3/anti-CD28 for 24 h, which was selected as a polyclonal stimulant due to its T-cell activation capabilities (21, 22). Supernatants were collected, centrifuged, and stored in a -80°C refrigerator until analysis. Later, the supernatants were shipped inside dry ice containers to the Swiss Institute of Allergy and Asthma Research (SIAF), Davos, Switzerland. Upon arrival, the samples were still frozen and stored in -80°C until analysis. Samples were defrosted right before the analyses and analyzed with Millipore HCYTOMAG-60K-36 and HCYP2MAG-62K-20 assay (Merck KGaA, Darmstadt, Germany) using the Bio-Plex 200 System utilizing the Bio-Plex Manager 6.0 Software (Bio-Rad, Cressier, Switzerland) to perform profiling of 56 different cytokines (**Supplementary Tables S1–S3**). Internal quality controls for all analytes were satisfactory. However, due to the limitations of quantitative multiplex ELISA profiling, a few cytokines did not reach the quantitative limit of detection (i.e., fluorescence was under or exceeded the quantification limit of the assay) (**Supplementary Table S3**). Each cytokine found in more than 50% of the patient samples within the limit of quantification was included for analyses (29/56, 52%), thus ensuring that conclusions would not be based on a minority of samples. Samples under the limit of detection were assigned half the value of the lower threshold of the assay (23, 24), and samples exceeding the limit of detection were set to the upper threshold of the assay (**Supplementary Tables S1, S2**) (25). A more detailed version of PBMC processing and cytokine analyses is provided in the **Supplementary Material**. The minimum and maximum values of the cytokines are shown in **Supplementary Tables S1, S2**.

### Statistics

The normality of the data distribution was tested using the Kolmogorov–Smirnov test. Due to the skewness of the data, cytokine levels were log_10_ or x² transformed when appropriate. For other statistics, when appropriate, we used the two-sample t-test, Mann–Whitney U test, χ^2^ test, Fisher exact test (when cell counts were <5), multivariable linear model analysis [in using the backward stepwise method to adjust for baseline differences, only statistically significant variables (*P* <.05) were included in the final model], Kruskal–Wallis H test, and negative binominal regression (JMP version 13.1.0, SAS Institute, Cary, NC, USA). A more detailed version of the statistics is provided in the **Supplementary Material**.

## Results

### Study population

Originally, 125 children were eligible for the Vinku2 study, among whom 12 declined to continue and 113 were enrolled for clinical follow-up. For the current study, all other than sole RV- or RSV-affected children were excluded before further analyses. Of the 113 enrolled children, 63 were eligible for PBMC analysis in the acute phase, and ultimately, cytology was done in 56 children. In the convalescent phase, 22 were excluded from further analyses due to randomization to prednisolone. Thus, 34 children were eligible in the convalescent phase at 2 weeks, and ultimately, cytology was done in 30 children. On further study points at 2 and 12 months, 30 children had clinical data available (**Figure 1**).

### Patient characteristics

The mean age of the study subjects was 12.5 months [interquartile range (IQR) 7.4–15.9], 69% were boys, 80% were treated as inpatients, 29% were atopic, and 20% had atopic eczema. Children infected with RV were older and heavier, had a higher blood eosinophil count, and had fewer preceding symptoms (wheezing, cough, rhinitis, fever) (all *P* <.05, **Table 1**). Due to these differences, the analyses comparing cytokine response from PBMCs were adjusted to the aforementioned variables using backward stepwise regression.

**Table 1:** Patient characteristics at study entry.

Characteristics	Rhinovirus (n = 47)	RSV (n = 9)	*P*-value
Age, months	13.5 (8.8–16.8)	6.1 (4.3–12.4)	**.006**
Male sex, no.	35 (74%)	4 (44%)	.11
Weight, kg	10.3 (2.1)	7.9 (1.2)	**.001**
Preceding wheezing, days	1 (1–1)	2 (1–3)	**.003**
Preceding cough, days	2 (2–3)	6 (3–7)	**.002**
Preceding rhinitis, days	3 (2–5)	5 (5–7)	**.005**
Preceding temperature over 37.5°C	1 (0–2)	2 (1–4)	**.002**
Clinical score, points	5 (4–8)	4 (2–8)	.31
Oxygen saturation, %	97 (95–98)	98 (96–98)	.27
Temperature, °C	37.4 (37.0–37.8)	37.1 (36.8–38.0)	.29
CRP, mg/L	13 (6–21)	4 (0–39)	.16
Eczema, no.	10 (22%)	1 (11%)	.53
Dr-dg atopic eczema, no.	10 (22%)	1 (12%)	.53
B-Eos (1 × 10^9^/L)	0.52 (0.35–0.73)	0.08 (0.04–0.17)	**<.001**
B-Eos >0.4 × 10^9^/L	29 (64%)	1 (11%)	**<.001**
Sensitization, no.	15 (33%)	1 (13%)	.41
Food, no.	14 (30%)	1 (13%)	.42
Aero, no.	10 (22%)	0 (0%)	.33
Perennial, no.	9 (20%)	0 (0%)	.33
Parental asthma, no.	9 (19%)	0 (0%)	.33
Parental allergy, no.	29 (62%)	3 (33%)	.15
Parental smoking, no.	22 (47%)	3 (33%)	.72
Virus load, copies/ml	5,100 (590–21,000)	No data	
S-25-OHD, nmol/L	84 (72–99)	78 (73–103)	0.94
S-25-OHD_2_, nmol/L	16 (0–30)	33 (18–56)	**0.04**
S-25-OHD_3_, nmol/L	65 (43–80)	57 (26–69)	.18

RSV, respiratory syncytial virus; Dr-dg, doctor-diagnosed; B-Eos, blood eosinophil count; S-25-OHD, serum 25-hydroxyvitamin D.

Values are shown as mean (SD), median (interquartile range), or number (%).

Data were analyzed by two-sample t-test, Mann–Whitney U-test, χ^2^ test, or Fisher exact test.

Bold text, statistical significance P <.05.

### Differences in cytokine expression at study entry

**Figure 2:**
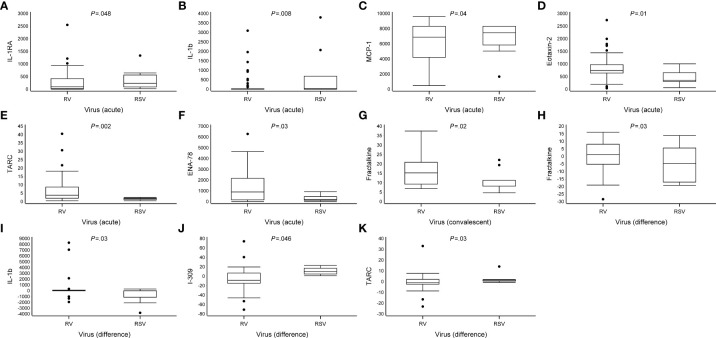
Differences in cytokine expression levels at study entry and convalescent phases. Data are presented as median [the lower (Q1) and upper (Q3) quartiles, and data falling outside the Q1–Q3 range are plotted as outliers]. Cytokine concentrations are presented as pg/ml. In the difference in the cytokine expression, multiple significant differences were observed between virus groups (RV vs. RSV, all *P* <.05) **(A–K)**.

Marked differences were observed in the cytokine response from PBMCs between the RV and RSV groups in response to anti-CD3/anti-CD28 stimulation. In adjusted analyses, when compared to the RSV group, the RV group was characterized by a lower expression of interleukin 1 receptor antagonist (IL-1RA) (median 240 vs. 97 pg/ml), interleukin 1 beta (IL-1β) (30 vs. 3.5), and monocyte chemoattractant protein-1 (MCP-1) (7,500 vs. 6,900) and a higher expression of eotaxin-2 (350 vs. 740), thymus- and activation-regulated chemokine (TARC) (1.8 vs. 3.9), and epithelial-derived neutrophil-activating peptide 78 (ENA-78) (210 vs. 900) during the acute phase (all *P* <.05; **Figure 2**, **Table 2** and **Supplementary Table S4**). Differences were also found in the expression of IL-6, I-309, and eotaxin-3, but significance was not reached (all.05 < *P* <.08; **Figure 2**, **Table 2** and **Supplementary Table S4**). The biological mechanisms of these cytokines are described in **Supplementary Table S5**.

**Table 2:** Differences in cytokine expression levels at study entry and the convalescent phase.

Cytokine	Timing	RV n (acute) = 47 n (convalescent) = 20n (difference) = 17	RSV n (acute) = 9n (convalescent) = 10n (difference) = 8	*P*-value univariate	*P*-value multivariate	Adjustments
Fractalkine	Acute Convalescent Difference	14 (8.3–28) 15 (9.3–21) 1.1 (-6.4–8.2)	13 (8.3–24) 8.3 (7.4–13) -4.6 (-17–8.4)	.46 **.03** .35	.73 **.02** **.03**	4 - 1,3
IL-1RA	Acute Convalescent Difference	97 (28–420) 170 (50–340) 24 (-280–200)	240 (78–600) 140 (15–320) -56 (-560–280)	.23 .57 .82	.**048** .98 .36	1 3 -
IL-1β	Acute Convalescent Difference	3.5 (1.6–25) 25 (1.6–270) 5.0 (0–220)	30 (1.6–1,400) 4.2 (1.6–120) -6.8 (-1,600–24)	.13 .32 .13	**.008** .27 **.03**	1 - -
IL-6	Acute Convalescent Difference	32 (6.8–380) 66 (4.2–2,000) 2.4 (-31–2,000)	110 (17–2,100) 29 (9.5–960) -61 (-2,100–28)	.28 .68 .10	.056 .70 .06	1 - -
MCP-1	Acute Convalescent Difference	6,900 (4,200–8,300) 6,700 (4,300–8,300) 0 (-3,800–2,700)	7,500 (5,400–8,300) 6,700 (4,900–7,700) -160 (-2,200–1,100)	.60 .93 .68	.**04** .09 .55	1 1,2,5 1,2
Eotaxin-2	Acute Convalescent Difference	740 (640–980) 770 (450–1,100) -52 (-340–320)	350 (180–740) 520 (330–980) 37 (-140–290)	**.01** .25 .56	**.01** .81 .36	- 1,2,5 1,2
I-309	Acute Convalescent Difference	32 (19–67) 24 (14–41) -8.4 (-20–7.8)	12 (7.8–21) 29 (11–34) 10 (3.8–17)	**.001** .68 **.02**	.08 .58 **.046**	2 5 -
TARC	Acute Convalescent Difference	3.9 (2.0–8.9) 4.4 (2.4–7.8) -0.96 (-2.4–2.5)	1.8 (0.88–2.1) 2.9 (1.9–3.6) 1.4 (0.28–1.9)	**.002** .31 .11	.**002** .64 .**03**	- 6,7 -
Eotaxin-3	Acute Convalescent Difference	110 (110–220) - -	110 (110–160) - -	.28 - -	.07 - -	7 - -
ENA-78	Acute Convalescent Difference	900 (170–2,200) 190 (65–1,800) -160 (-890–68)	210 (73–470) 230 (50–1,100) -30 (-200–440)	**.02** .86 .20	**.03** .65 .32	- 1,2,5 2

Acute sample, samples drawn at study entry; Convalescent sample, samples drawn at the 2-week follow-up; Difference, difference in cytokine expression when comparing samples drawn at the 2-week follow-up and at study entry.

Values are shown as medians (interquartile range).

Data were analyzed by Mann–Whitney U-test, and by multivariable linear model analysis (after log- or x²-transformation). The adjustments for immunologic analyses included baseline characteristics that significantly differed between the groups [Age = 1, weight = 2, duration of previous symptoms (rhinitis = 3, cough = 4, wheezing = 5, fever = 6), and B-Eos = 7 at entry]. A backward stepwise method was used for the final adjustment model separately for each cytokine. Only statistically significant variables (P <.05) were included in the final model.

All data are shown in **Supplementary Table S4**.

Bold text, statistical significance P <.05.

### Differences in cytokine expression at the convalescent phase

In the convalescent phase (2 weeks later), in adjusted analyses, the cytokine profile of the RV group was characterized by a higher expression of fractalkine when compared to the RSV group (median 15 vs. 8.3 pg/ml, *P* = .02; **Table 2**, **Figure 2** and **Supplementary Table S4**). An analysis of the change in cytokine expression between the acute and convalescent phases revealed an increased expression of fractalkine (median 1.1 vs. -4.6 pg/ml) and IL-1β (5.0 vs. -6.8, respectively) in the RV group, whereas in the RSV group, the expression was decreased (all *P <*.03). Moreover, the RV group was characterized by a decreased expression of I-309 (median -8.4 vs. 10 pg/ml) and TARC (-0.96 vs. 1.4), whereas in the RSV group, the expression was increased (all *P* <.05; **Figure 2**, **Table 2** and **Supplementary Table S4**). A difference in the change in cytokine secretion was also observed with IL-6, but it did not reach significance (*P* = .06).

### The association between the cytokine expression and the severity of acute illness

Considering the duration of hospitalization, several statistically significant interactions between the virus group and cytokine response were observed (all *P* <.04; **Table 3** and **Supplementary Table S6**), indicating that the effect of cytokine response from PBMCs was different in the RV and RSV groups in hospitalization time. Increased expression of interferon gamma (IFN-γ), macrophage-derived chemokine (MDC), IL-1RA, and vascular endothelial growth factor (VEGF) was associated with shorter hospitalization times in the RSV group (all *P* <.02), but in the RV group, this difference was not significant (all *P >*.69; **Table 3** and **Supplementary Table S6**). A significant virus group × cytokine expression interaction was observed with IL-6, but the expression of IL-6 was not associated with the duration of hospitalization in the RSV or RV group (all *P* >.08).

**Table 3:** Association between cytokine expression and severity of acute illness (duration of hospitalization).

Cytokine		Group effect RV vs. RSV		Cytokine effect Expression of cytokine		Group × cytokine interaction effect
		Estimate (95% CI)	*P*	Estimate (95% CI)	*P*	*P*
IFN-γ		‡	‡	1.033‡ (0.878, 1.216) 0.651† (0.460, 0.922)	.69^§^ .**02** ^#^	.**03**
MDC		‡	‡	1.047‡ (0.841, 1.304) 0.609† (0.428, 0.868)	.68^§^ .**001** ^#^	.**02**
IL-1RA		‡	‡	1.026‡ (0.825, 1.276) 0.481† (0.289, 0.801)	.82^§^ .**005** ^#^	.**02**
IL-6		‡	‡	1.088‡ (0.938, 1.262) 0.764† (0.566, 1.032)	.26^§^ .08^#^	.**04**
VEGF		‡	‡	1.190* (0.940, 1.510) 0.385† (0.290, 0.530)	.16^§^ <.**0001** ^#^	.**0004**

Data were analyzed by negative binominal regression with log-transformed cytokine level.

CI, confidence interval.

^*^Relative risk, RV group negative binomial regression.

^†^Relative risk, RSV group negative binomial regression.

^§^Group effect in the RV treatment arm.

^#^Group effect in the placebo treatment arm.

‡Due to the significant interaction, the cytokine effect was not estimated using all data. The effect of cytokine is presented separately in the RV and RSV groups.

All data are shown in **Supplementary Table S6**.

Bold text, statistical significance P <.05.

### The association between the cytokine expression and recurrences and asthma

**Figure 3:**
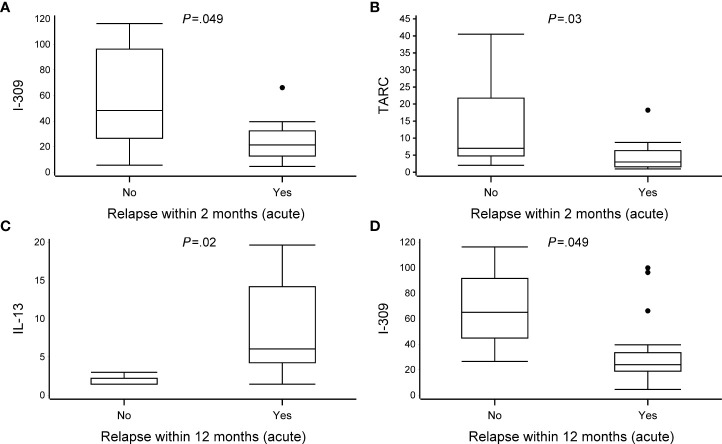
The association between cytokine expression and recurrences at 2 and 12 months. Data are presented as median [the lower (Q1) and upper (Q3) quartiles, and data falling outside the Q1–Q3 range are plotted as outliers]. Cytokine concentrations are presented as pg/ml. Due to scarcity of the occurrence of relapses in the RSV group, only patients from the RV group were included in the analyses. In the association between cytokine expression and recurrences at 2 and 12 months, multiple significant differences were observed between virus groups (relapse vs. no-relapse, all *P* <.05) **(A–D)**. An analysis of difference was excluded due to the small number of patients in the no-relapse group (n = 3). All data are shown in **Supplementary Table S7**.

Although the incidence of relapses within 2 and 12 months differed between the RV and RSV groups [52% vs. 11% (*P* = .02) and 81% vs. 22% (*P* = .002), respectively], the exact cytokine response for this difference remained concealed because of the scarcity of the occurrence of relapses in the RSV group. However, in the RV group, a decreased expression of I-309 (CCL1) and TARC during the acute phase was associated with the occurrence of a new physician-confirmed wheezing episode within 2 months (median, relapse vs. no relapse, 21 vs. 48, *P* = .049, and 3.0 vs. 7.0, *P* = .03, respectively). Moreover, in the acute phase, an increased expression of IL-13 (6.0 vs. 1.5) and a decreased expression of I-309 (CCL1, 24 vs. 65) were associated with the occurrence of a new physician-confirmed wheezing episode within 12 months in the RV group (all *P* <.05; **Figure 3**, **Table 4** and **Supplementary Table S7**). Overall, in the RV group and in the RSV group, due to the limited number of children, the association of the cytokine expression with 4-year asthma could not be assessed (data not shown).

**Table 4:** The association between cytokine expression and recurrences.

		2 months			12 months			
Cytokine	Timing	No relapsen (acute) = 10n (convalescent) = 10	Relapsen (acute) = 11n (convalescent) = 10	*P*-value	No relapsen (acute) = 4n (convalescent) = 4	Relapsen (acute) = 17n (convalescent) = 16	*P*-value
Fractalkine	Acute Convalescent	14 (8.3–30) 16 (8.0–24)	14 (8.3–28) 13 (9.3–20)	.89 .73	9.5 (5.9–25) 570 (120–1,850)	17 (8.3–28) 13 (8.5–20)	.28 .07
IFN-γ	Acute Convalescent	9.3 (1.5–51) 63 (1.6–400)	14 (1.5–81) 8.0 (1.6–410)	.91 .54	1.5 (1.5–100) 320 (32–1,400)	14 (1.5–53) 8.0 (1.6–350)	.39 .07
IL-13	Acute Convalescent	5.3 (1.5–16) 4.7 (1.5–24)	5.0 (1.5–9.4) 3.2 (1.5–33)	.97 .85	1.5 (1.5–2.6) 12 (3.5–42)	6.0 (3.6–17) 3.0 (1.5–7.6)	**.02** .31
IL-1β	Acute Convalescent	1.6 (1.6–1,100) 63 (1.6–2,000)	1.6 (1.6–25) 20 (1.6–500)	.74 .51	1.6 (1.6–2,300) 220 (40–6,300)	1.6 (1.6–170) 12 (1.6–92)	.84 .09
IL-6	Acute Convalescent	13 (3.7–2,000) 300 (3.4–2,000)	55 (5.4–280) 66 (4.1–2,400)	.89 .82	7.8 (2.3–1,500) 2,000 (510–7,200)	55 (5.9–1,100) 54 (2.2–1,300)	.42 .09
MCP-1	Acute Convalescent	6,400 (3,900–8,300) 4,700 (2,500–8,300)	7,500 (4,900–8,300) 7,900 (6,200–8,300)	.47 .09	4,200 (2,200–7,500) 6,500 (4,700–8,300)	7,500 (5,300–8,300) 6,700 (3,200–8,300)	.11 .70
MIP-1α	Acute Convalescent	70 (27–900) 430 (48–1,100)	210 (120–1,400) 450 (41–1,400)	.12 .94	27 (19–1,000) 430 (390–1,920)	200 (70–1,200) 430 (32–1,300)	.06 .45
I-309	Acute Convalescent	48 (26–97) 23 (12–43)	21 (13–32) 24 (16–42)	**.049** .88	65 (36–100) 16 (11–42)	24 (16–36) 26 (17–41)	**.049** .40
IL-16	Acute Convalescent	78 (48–130) 44 (33–75)	70 (49–81) 53 (38–77)	.52 .60	79 (49–140) 34 (30–44)	70 (48–87) 54 (42–83)	.57 .07
TARC	Acute Convalescent	7.0 (4.1–26) 4.0 (2.2–12)	3.0 (1.6–6.3) 4.1 (2.0–7.4)	**.03** .65	5.9 (2.7–32) 2.6 (2.4–56)	4.0 (1.9–8.7) 5.2 (2.0–7.8)	.45 .85

Acute, samples drawn at study entry; Convalescent, samples drawn at the 2-week follow-up.

Values are shown as medians (interquartile range).

Data were analyzed by Mann–Whitney U-test.

Analysis of difference was excluded from the table due to the small number of patients in the no-relapse group (n = 3). All data are shown in **Supplementary Table S7**.

Bold text, statistical significance P <.05.

### The association between the cytokine expression and Rhinovirus species and genome load

No statistically significant differences were found in the cytokine expression between different RV species or according to the RV genome load (data not shown).

## Discussion

To our knowledge, this study is the first to simultaneously analyze multiple cytokines from stimulated PBMCs in young first-time wheezing children infected with sole RV or sole RSV. Our main findings were that 1) there are distinct differences between cytokine responses from PBMCs and RV- and RSV-induced first wheezing episode, especially in the acute phase; 2) there is an association between the cytokine response from PBMCs and the severity of acute illness between the two virus groups; and 3) specific cytokine responses from PBMCs were associated with medium-term prognosis in the RV group.

Although the RV and RSV groups shared similarities in overall cytokine expression, acute-phase samples from the RV group were more of the Type 2 subtype, whereas in comparison, acute-phase samples from the RSV group were more of the Type 1 subtype as well as proinflammatory-associated cytokine profiles. Different types of cell-mediated immunity have been previously described in detail (26). At study entry, children infected with RV were characterized by a higher expression of eotaxin-2 and TARC; the former promotes the migration of eosinophils into the lungs (27), and the latter selectively binds to CCR4, leading to the activation of a type 2 immune response *via*, e.g., Th2 cells, ILC2, and airway eosinophils (28). The expression of ENA-78, although counterintuitive is primarily a neutrophil chemoattractant, was higher in children infected with RV (29). Children infected with RSV were characterized by a higher activity of proinflammatory-associated cytokines IL-1β and its antagonist IL-1RA. Of note, RSV-affected children were characterized by a higher expression of MCP-1, which is induced *via* alveolar epithelial damage and has a wide range of immunological functions such as immediate neutrophil recruitment and recruitment of fibrocytes and profibrotic macrophages as well as Th cell polarization (30, 31). However, MCP-1 is associated with both type 1 and type 2 immunity depending on environmental factors, such as tissue site, type of pathogen, and induction timing (32). Moreover, the macrophage polarization is regulated by the MCP-1–CCR2 axis, and blocking MCP-1 might lead to the upregulation of M1 polarization-associated genes (33).

In the convalescent phase, the former difference was more balanced. Only the expression of fractalkine was increased in the RV group when compared to the RSV group. These results are in line with findings from previous studies on nasopharyngeal aspirates and serum samples (34–36). Furthermore, regarding the change in the cytokine response from PBMCs, the RV group was characterized by a decreasing trend of Type 2-associated profile (I-309 and TARC) and an increasing trend of Type 1- and Th17-associated profiles (fractalkine and IL-1β, respectively). Although previous studies (from nasal swabs) have shown differences in IFN-γ and IL-10 expression between children infected with RV and RSV (37), one recent study showed that this difference dissipated when RV-bronchiolitis and RSV-bronchiolitis are accompanied by wheezing (38). This finding is in line with our results.

Interestingly, the cytokine response was not associated with the severity of illness in the RV group. However, in the RSV group, a higher expression of IFN-γ, MDC, IL-1RA, and VEGF was associated with a shorter duration of hospitalization, of which, a lower IFN-γ response has previously been shown to be associated with a more severe clinical course (39). Of note, these cytokines are classified into a wide range of functional groups [IFN-γ, Type 1 subtype; MDC, Type 2 subtype; IL-1RA, proinflammatory activity; VEGF, regulatory T (Treg) cell activity], and these cytokines, for example, MDC (40), have overlapping properties (Type 1 and Type 2 subtypes as well as Treg).

According to a recent meta-analysis, RV-induced early wheezing has been shown to be more strongly associated with and a major risk factor for subsequent relapse or asthma when compared to RSV (6). Although data concerning the cytokine expression and long-term prognosis in young wheezing children are scarce, one study suggested that increased expression of macrophage inflammatory protein-1 alpha (MIP-1α) was associated with recurrences. However, the study did not separate viral etiologies (41). Another study on RSV-affected children showed that a decreased expression of tumor necrosis factor-alpha (TNF-α) was associated with recurrences (42). However, to our knowledge, no prior study has studied this setting in first-time RV-affected wheezing children, who are by the current knowledge at greatest risk of recurrences and development of asthma (6). In our data, increased expression of I-309 (CCL1) and TARC in the RV group was associated with fewer relapses within 2 months. Additionally, a decreased expression of IL-13 and an increased expression of I-309 (CCL1) were associated with fewer relapses within 12 months. Interestingly, a change in cytokine expression was also associated with relapse within 2 (IFN-α2) and 12 months [granulocyte colony-stimulating factor (G-CSF), fractalkine, IL-1RA, IL-1β, IL-6, MCP-1], suggesting that inadequate timing of cytokine expression might mitigate improper clearance of viral inflammation (**Supplementary Table S7**). However, the sample size at the 12-month follow-up for difference analysis in the no-relapse group was relatively small, and therefore, the corresponding results should be considered hypothesis-generating only.

The RV-induced wheezing illness has many asthma-like characteristics both clinically (dry cough, wheezing) and pathophysiologically (Type 2 subtype polarized immune response and pronounced atopic characteristics) (1). Although the expression of cytokines shared similarities, RV seemed to trigger more Type 2 subtype cytokine profile compared to RSV. Surprisingly, in the convalescent phase, this difference appeared to dissipate, and, ultimately, the remaining difference in the RV group was a higher expression of fractalkine, which induces chemotaxis and has antiviral properties (43). This finding is in line with the clinical phenotypes of RV- and RSV-induced bronchiolitis. The biological mechanisms of all significant cytokines are presented in **Supplementary Table S5**.

Although previous studies have observed differences in the cytokine expression between RV serotypes or RV genome loads (44), in our study, this difference remained concealed. This difference is possible due to differences in stimulation protocols and might be duplicable only under a similar stimulation protocol. Of note, the association of virus load with the severity of illness might be age-dependent (45). Moreover, although anti-CD3/anti-CD28 closely mimics physiological T-cell receptor (TCR)-mediated T-cell activation by antigen-presenting cells (46), different stimulation settings might present altered cytokine responses. Therefore, it is difficult to compare the results from our study with studies conducted with different stimulation protocols; our findings should be confirmed with those of similar settings. Interestingly, not all significantly different cytokines were T cell-derived. However, although stimulation of PBMCs with anti-CD3/anti-C28 activates T cells directly, it may activate other classes of lymphocytes indirectly, resulting in increased levels of non-T cell-derived cytokines (47). Our cytokine panels were broad, capable of measuring multiple inflammatory events, not just T-cell responses. However, this was partly intentional, since the study design is novel, and thus, we did not have a hypothesis of which responses and differences to anticipate.

The strengths of the current study included detailed viral diagnostics, careful characterization of the subjects, and a detailed prospective follow-up in the original trial, as well as comprehensive analyses of cytokine profiles. Our original hypothesis was to differentiate two diseases from one other, hence the absence of a “control” group. However, our study has some limitations. First, statistical power analyses were not performed, and the rather small sample size did not permit optimal analyses in the multivariable model. However, both study groups were composed of carefully characterized novel bronchiolitis subgroups. Second, a small volume of culture medium limited the ability to perform optimal dilution series, and the fluorescence of some of the cytokines exceeded the limit of quantification and therefore complicated the analyses for some cytokines. Furthermore, the number of affected cytokines was relatively low (**Supplementary Table S3**). Third, our results may not be generalizable to outpatients, since the majority (80%) of the subjects were enrolled from hospital wards and the sample size was too small to permit a meaningful analysis of inpatient vs. outpatient interactions. Lastly, the cytokine response from stimulated PBMCs may not reflect the response in the lower airways, and the study results may be generalizable to moderate-to-severe wheezing children only.

In summary, our current study and earlier trials support the emerging assumption that RV- and RSV-induced wheezing illnesses differentiate from each other at multiple levels—from clinical manifestation to cellular responses manifested by altered cytokine and chemokine profiles. Our findings also reveal new and early potential biomarkers for short- and medium-term prognoses in high-risk cohorts, mainly RV- or RSV-affected first-time wheezing children. However, further trials are warranted.

## Data availability statement

The raw data supporting the conclusions of this article will be made available by the authors, without undue reservation.

## Ethics statement

The studies involving human participants were reviewed and approved by Ethics Committee of Turku University Hospital. Written informed consent to participate in this study was provided by the participants’ legal guardian/next of kin.

## Author contributions

The study protocol and manuscript were written by the investigators. Data were collected by study physicians (TJ, RT, AL) and analyzed by investigators (PH and TJ), and while consulting statistician (TV). MK, MT, and PH performed literature search and reviewed previous trials. Viral analyses were supervised by the TV, participated in drafting the original study protocol, providing primers for rhinovirus detection, and writing the manuscript. BR, MA, and CA supervised the cytokine analyses. All authors were involved in writing or reviewing the manuscript.

## Funding

Supported by the Academy of Finland (grant numbers 132595 and 114034), Helsinki; Finnish Medical Foundation, Helsinki; the Sigrid Jusélius Foundation, Helsinki; the Foundation for Pediatric Research, Helsinki; the Finnish Cultural Foundation, Turku and Helsinki; Turku University Foundation, Turku; the Paulo Foundation, Helsinki; and the Allergy Research Foundation, Helsinki – all in Finland. The study was supervised by TJ Leiras Takeda (Helsinki, Finland) who provided prednisolone and placebo preparations, but did not offer any financial support nor required any confidentiality agreements. The granting agencies covered all costs and played no role in the study design, data analysis, or manuscript preparation.

## Conflict of interest

The authors declare that the research was conducted in the absence of any commercial or financial relationships that could be construed as a potential conflict of interest.

## Publisher’s note

All claims expressed in this article are solely those of the authors and do not necessarily represent those of their affiliated organizations, or those of the publisher, the editors and the reviewers. Any product that may be evaluated in this article, or claim that may be made by its manufacturer, is not guaranteed or endorsed by the publisher.

## Abbreviations

ENA-78, epithelial-derived neutrophil-activating peptide 78, CXCL5; eotaxin-2, eosinophil chemotactic protein 2, CCL24; eotaxin-3, eosinophil chemotactic protein 3, CCL26; fractalkine, CX3CL1; I-309, CCL1; IFN-γ, interferon gamma; IL-1β, interleukin 1 beta; IL-1RA, interleukin 1 receptor antagonist; IL-6, interleukin 6; IL-13, interleukin 13; MCP-1, monocyte chemoattractant protein-1, CCL2; MDC, macrophage-derived chemokine, CCL22; MIP-1α, macrophage inflammatory protein-1 alpha, CCL4; TARC, thymus- and activation-regulated chemokine, CCL17; VEGF, vascular endothelial growth factor.
