# Supplementary material for: Cytokine expression in rhinovirus- vs. respiratory syncytial virus-induced first wheezing episode and its relation to clinical course
Source: Front Immunol. 2022 Nov 14;13:1044621. doi: 10.3389/fimmu.2022.1044621 (PMC9702984; doi:10.3389/fimmu.2022.1044621)
Supplement: Supplementary file 1 [file Table_1.docx]

**Supplementary Table 1:** Minimum limits of quantification of the cytokine plates (pg/ml)

| Cytokine plate (LLOQ) | Median | IQR | SEM |
| --- | --- | --- | --- |
| EGF | 3,201 | 3,199-3,2085 | 0,0013775 |
| FGF-2 | 20,717 | 15,6135-79,999 | 7,2094411 |
| Eotaxin | 3,245 | 3,229-3,278 | 0,0093527 |
| TGF-a | 3,198 | 3,197-3,2 | 0,0016662 |
| G-CSF | 17,707 | 3,956-47,5715 | 6,3179238 |
| GM-CSF | 3,189 | 3,04925-3,23425 | 0,0547066 |
| Fractalkine | 16,502 | 13,3345-18,3885 | 1,0211085 |
| IFNa2 | 2,924 | 2,7215-3,075 | 0,0543252 |
| IFNg | 3,22 | 3,191-3,2625 | 0,0133769 |
| IL-10 | 3,199 | 3,188-3,211 | 0,0099863 |
| MCP-3 | 3,358 | 3,2795-3,4825 | 0,6124192 |
| IL-12P40 | 3,128 | 3,0825-3,17 | 0,0160411 |
| MDC | 3,231 | 3,2135-3,277 | 154,22677 |
| IL-12P70 | 3,238 | 3,2055-3,253 | 0,0081075 |
| IL-13 | 2,898 | 2,7565-3,083 | 0,0578263 |
| IL-15 | 3,101 | 3,033-3,1285 | 0,0120434 |
| sCD40L | 2,844 | 2,6685-3,1015 | 0,8526815 |
| IL-17A | 2,824 | 2,627-3,0255 | 0,0596661 |
| IL-1RA | 2,988 | 2,901-3,134 | 0,0354376 |
| IL-1a | 3,207 | 3,1855-3,2385 | 0,0096655 |
| IL-9 | 3,204 | 3,178-3,224 | 0,0076101 |
| IL-1b | 3,196 | 3,174-3,2235 | 0,0069012 |
| IL-2 | 3,179 | 3,1595-32535 | 0,0148749 |
| IL-3 | 3,205 | 3,192-3,2215 | 0,0059313 |
| IL-4 | 3,974 | 2,565-17,905 | 4,8007929 |
| IL-5 | 3,215 | 3,116-3,2315 | 0,0136184 |
| IL-6 | 3,173 | 3,155-3,2175 | 0,0139667 |
| IL-7 | 2,962 | 2,8655-3,171 | 0,0823975 |
| IL-8 | 3,193 | 3,1735-3,2045 | 0,0048934 |
| IP-10 | 15,262 | 13,9445-19,5485 | 4,3331332 |
| MCP-1 | 3,221 | 3,2135-3,2395 | 0,0078529 |
| MIP-1a | 3,123 | 3,0885-3,1785 | 0,0107553 |
| MIP-1b | 3,152 | 2,838-17,261 | 1,4759952 |
| RANTES | 3,024 | 2,8815-3,127 | 0,0381726 |
| TNFa | 3,203 | 3,1565-3,2415 | 0,0109037 |
| VEGF | 421,913 | 47,8025-1516,0325 | 160,94344 |
| Eotaxin-2 | 9,78 | 9,725-9,837 | 0,0163266 |
| MCP-2 | 4,884 | 4,857-4,887 | 0,0099127 |
| MCP-4 | 9,662 | 9,615-9,7525 | 0,0269650 |
| I-309 | 8,553 | 2,2285-9,228 | 0,7707900 |
| IL-16 | 9,44 | 8,577-10,0975 | 6,8821392 |
| TARC | 0,977 | 0,9735-0,979 | 0,0013691 |
| Eotaxin-3 | 226,945 | 116,412-252,829 | 122,96209 |
| LIF | 19,489 | 19,218-19,709 | 0,0609256 |
| TPO | 48,485 | 47,772-49,612 | 0,2892089 |
| SCF | 9,618 | 9,53-9,8675 | 0,0608072 |
| TSLP | 9,777 | 9,7655-9,7925 | 0,0087902 |
| IL-33 | 19,519 | 19,44-19,5555 | 0,0386083 |
| IL-20 | 48,76 | 47,874-49,7495 | 0,3012482 |
| IL-21 | 19,509 | 19,489-19,543 | 0,0505206 |
| IL-23 | 47,233 | 44,342-48,999 | 11,967690 |
| TRAIL | 9,725 | 9,556-9,771 | 0,0530875 |
| SDF-1a+b | 96,157 | 95,16-98,0375 | 0,6423440 |
| ENA-78 | 19,532 | 19,4115-19,5795 | 0,0279694 |
| MIP1-d | 48,843 | 48,827-48,8928 | 0,0118887 |
| IL-28A | 9,315 | 8,5575-9,717 | 0,1801218 |

Values shown as median (pg/ml), IQR (pq/ml), and SEM (pg/ml)

LLOQ, lower limit of quantification; IQR, interquartile range; SEM, standard error of the mean

All Elisa cytokines analyze plates, and their lower limits of detection and plate variation are included.

**Supplementary Table 2:** Maximum limits of quantification of the cytokine plates (pg/ml)

| Cytokine plate (ULOQ) | Median (pg/ml) | IRQ (pq/ml) | SEM |
| --- | --- | --- | --- |
| EGF | 2381,015 | 1015,2425-8362,5905 | 859,46008 |
| FGF-2 | 10004,972 | 9955,22-10062,4615 | 43,952348 |
| Eotaxin | 378,452 | 371,28-383,1285 | 185,56257 |
| TGF-a | 451,888 | 422,4575-1529,9865 | 640,91664 |
| G-CSF | 10001,761 | 9999,93-10008,9825 | 7,0598313 |
| GM-CSF | 10042,933 | 9943,88-10128,525 | 44,343921 |
| Fractalkine | 10028,752 | 99964,6155-10053,1905 | 20,379075 |
| IFNa2 | 10009,96 | 10000,854-10031,0765 | 21,363853 |
| IFN-g | 9995,598 | 9977,161-10001,277 | 15,676256 |
| IL-10 | 10002,533 | 9989,1795-10049,234 | 23,650620 |
| MCP-3 | 390,511 | 384,236-394,5475 | 61,961892 |
| IL-12P40 | 10152,93 | 10052,021-10193,977 | 22,448075 |
| MDC | 9307,716 | 8671,246-9578,7925 | 168,99341 |
| IL-12P70 | 10181,091 | 10071,225-10323,799 | 42,26238 |
| IL-13 | 10006,142 | 10001,036-10015,6515 | 4,3750578 |
| IL-15 | 10139,205 | 10081,423-10218,147 | 28,554315 |
| sCD40L | 9979,539 | 7841,006-10030,406 | 639,08309 |
| IL-17A | 10214,776 | 10054,0225-10302,2195 | 399,86539 |
| IL-1RA | 10051,847 | 10001,3985-10101,552 | 15,574531 |
| IL-1a | 9942,949 | 9712,9255-10072,156 | 127,68354 |
| IL-9 | 9748,562 | 8506,975-10986,7635 | 4062,1438 |
| IL-1b | 9958,633 | 9407,71-10534,795 | 425,40063 |
| IL-2 | 10069,675 | 9677,8875-10273,7055 | 120,03585 |
| IL-3 | 9922,288 | 9766,4375-10106,154 | 93,396526 |
| IL-4 | 9999,993 | 9999,977-10000,8155 | 11,281399 |
| IL-5 | 8087,257 | 2232,7605-9267,4985 | 844,19341 |
| IL-6 | 1961,226 | 1851,749-5090,467 | 681,33094 |
| IL-7 | 1956,798 | 1914,7035-1982,077 | 577,48361 |
| IL-8 | 8499,736 | 1832,494-9580,258 | 910,62747 |
| IP-10 | 10036,185 | 9859,8785-10430,457 | 395,83713 |
| MCP-1 | 8329,005 | 4948,357-8973,66 | 806,47168 |
| MIP-1a | 438,906 | 407,786-2148,262 | 197,02361 |
| MIP-1b | 10444,606 | 10155,399-11100,162 | 170,63137 |
| RANTES | 10397,108 | 5184,172-11031,538 | 881,27285 |
| TNFa | 9974,745 | 9541,3995-10576,118 | 231,76102 |
| VEGF | 10940,242 | 10276,847-11653,443 | 989,44725 |
| Eotaxin-2 | 1864,788 | 619,291-2448,318 | 497,07648 |
| MCP-2 | 1436,256 | 305,749-4109,2945 | 448,64692 |
| MCP-4 | 1865,255 | 652,057-3049,4671 | 795,85317 |
| I-309 | 2000,863 | 1018,939-2012,5245 | 141,60260 |
| IL-16 | 9393,522 | 2263,121-11466,92 | 975,06463 |
| TARC | 940,58 | 825,0265-1056,9115 | 76,057271 |
| Eotaxin-3 | 49963,518 | 37301,3625-50016,5245 | 3363,2400 |
| LIF | 1970,845 | 19779,106-20584,886 | 105,46329 |
| TPO | 50146,231 | 49693,747-51370,179 | 257,70843 |
| SCF | 10085,011 | 9991,9225-10134,9705 | 30,173207 |
| TSLP | 9877,377 | 9428,8745-10552,221 | 495,38582 |
| IL-33 | 15148,003 | 4256,699-18815,7295 | 1655,5968 |
| IL-20 | 48910,21 | 45178,4795-54632,824 | 2233,7796 |
| IL-21 | 18996,286 | 4161,226-22054,822 | 2057,4317 |
| IL-23 | 50041,13 | 50004,595-50264,9745 | 72,753,499 |
| TRAIL | 9907,719 | 9692,716-10120,549 | 114,39589 |
| SDF-1a+b | 97816,886 | 25678,9545-110043,545 | 8771,4732 |
| ENA-78 | 14497,787 | 4147,6415-17792,098 | 1590,1992 |
| MIP1-d | 3601,183 | 3140,6725-9289,259 | 2924,5720 |
| IL-28A | 10000,016 | 9997,632-10003,3665 | 428,69315 |

Values shown as median (pg/ml), IQR (pq/ml), and SEM (pg/ml)

ULOQ, upper limit of quantification; IQR, interquartile range; SEM, standard error of the mean

All Elisa cytokines analyze plates, and their upper limits of detection and plate variation are included.

**Supplementary Table 3:** The quantification of the cytokines

| Cytokine | Acute phase n=97 | | | | | Convalescent phase n=91 | | | | |
| --- | --- | --- | --- | --- | --- | --- | --- | --- | --- | --- |
|  | Within range | OOR < | OOR > | * | *** | Within range | OOR < | OOR > | * | *** |
| EGF | 55 (57%) | 17 |  | 25 |  | 32 (35%) | 21 |  | 38 |  |
| FGF-2 | 39 (40%) | 57 |  | 1 |  | 26 (29%) | 65 |  |  |  |
| Eotaxin | 12 (12%) | 46 |  | 39 |  | 11 (125) | 36 |  | 44 |  |
| TGF-a | 14 (14%) | 63 |  | 20 |  | 12 (13%) | 59 |  | 20 |  |
| G-CSF | 75 (77%) | 20 |  | 1 |  | 64 (70%) | 25 |  | 2 |  |
| GM-CSF | 53 (55%) | 10 |  | 23 | 11 | 45 (49%) | 20 |  | 15 | 10 |
| Fractalkine | 71 (73%) | 23 |  | 1 | 2 | 72 (79%) | 19 |  |  |  |
| IFNa2 | 73 (75%) | 9 |  | 14 | 1 | 47 (52%) | 26 |  | 18 |  |
| IFN-g | 57 (59%) | 12 |  | 28 |  | 52 (57%) | 19 |  | 20 |  |
| IL-10 | 85 (88%) | 1 |  | 11 |  | 75 (82%) | 3 |  | 13 |  |
| MCP-3 | 71 (73%) | 1 | 25 |  |  | 60 (66%) | 1 | 27 | 3 |  |
| IL-12P40 | 31 (32%) | 53 |  | 13 |  | 31 (34%) | 50 |  | 10 |  |
| MDC | 91 (94%) |  | 2 | 4 |  | 87 (96%) | 1 | 1 | 2 |  |
| IL-12P70 | 26 (27%) | 25 |  | 46 |  | 27 (30%) | 27 |  | 37 |  |
| IL-13 | 64 (66%) | 23 |  | 10 |  | 58 (64%) | 24 |  | 9 |  |
| IL-15 | 3 (3%) | 77 |  | 17 |  | 5 (5%) | 73 |  | 13 |  |
| sCD40L | 42 (43%) | 36 |  | 19 |  | 35 (38%) | 40 |  | 16 |  |
| IL-17A | 34 (35%) | 59 |  | 4 |  | 22 (24%) | 61 |  | 8 |  |
| IL-1RA | 95 (98%) | 1 |  | 1 |  | 85 (93%) | 2 |  | 4 |  |
| IL-1a | 36 (37%) | 40 |  | 21 |  | 37 (41%) | 42 |  | 12 |  |
| IL-9 | 6 (6%) | 62 | 1 | 28 |  | 6 (7%) | 57 |  | 28 |  |
| IL-1b | 53 (55%) | 10 |  | 34 |  | 46 (51%) | 12 |  | 32 |  |
| IL-2 | 51 (53%) | 21 |  | 25 |  | 46 (51%) | 30 |  | 15 |  |
| IL-3 | 15 (15%) | 45 |  | 37 |  | 10 (11%) | 46 |  | 35 |  |
| IL-4 | 42 (43%) | 44 |  | 11 |  | 38 (42%) | 47 |  | 6 |  |
| IL-5 | 22 (23%) | 32 |  | 43 |  | 18 (20%) | 28 |  | 45 |  |
| IL-6 | 72 (74%) | 10 | 7 | 7 | 1 | 51 (56%) | 16 | 7 | 17 |  |
| IL-7 | 43 (44%) | 37 |  | 15 | 2 | 33 (36%) | 44 |  | 14 |  |
| IL-8 | 91 (94%) |  | 5 | 1 |  | 86 (95%) |  | 3 | 2 |  |
| IP-10 | 71 (73%) | 1 | 16 | 9 |  | 73 (80%) |  | 11 | 7 |  |
| MCP-1 | 71 (73%) |  | 11 | 15 |  | 69 (76%) |  | 5 | 17 |  |
| MIP-1a | 79 (81%) | 1 | 5 | 12 |  | 71 (78%) |  | 4 | 16 |  |
| MIP-1b | 97 (100%) |  |  |  |  | 91 (100%) |  |  |  |  |
| RANTES | 97 (100%) |  |  |  |  | 90 (99%) |  |  | 1 |  |
| TNFa | 94 (97%) |  |  | 3 |  | 81 (89%) |  |  | 10 |  |
| VEGF | 54 (56%) | 32 |  | 11 |  | 45 (49%) | 32 |  | 14 |  |
| Eotaxin-2 | 97 (100%) |  |  |  |  | 89 (98%) | 1 |  | 1 |  |
| MCP-2 | 87 (90%) |  | 2 | 8 |  | 84 (92%) |  |  | 7 |  |
| MCP-4 | 46 (47%) | 2 |  | 45 | 4 | 30 (33%) | 7 |  | 53 | 1 |
| I-309 | 93 (96%) | 4 |  |  |  | 87 (96%) | 4 |  |  |  |
| IL-16 | 95 (98%) |  |  |  | 2 | 89 (98%) | 1 |  | 1 |  |
| TARC | 86 (89%) |  |  | 8 | 3 | 82 (90%) |  |  | 8 | 1 |
| Eotaxin-3 | 53 (55%) | 44 |  |  |  | 45 (49%) | 46 |  |  |  |
| LIF | 4 (4%) | 33 |  | 54 | 6 | 7 (8%) | 45 |  | 37 | 2 |
| TPO | 5 (5%) | 49 |  | 42 | 1 | 5 (5%) | 48 |  | 38 |  |
| SCF | 3 (3%) | 68 |  | 26 |  | 3 (3%) | 66 |  | 22 |  |
| TSLP | 2 (2%) | 54 |  | 39 | 2 | 1 (1%) | 53 |  | 37 |  |
| IL-33 | 1 (1%) | 51 |  | 44 | 1 | 1 (1%) | 53 |  | 37 |  |
| IL-20 | 38 (39%) | 28 |  | 29 | 2 | 37 (41%) | 29 |  | 24 | 1 |
| IL-21 | 1 (1%) | 70 |  | 23 | 3 | 1 (1%) | 66 |  | 21 | 3 |
| IL-23 | 20 (21%) | 51 |  | 26 |  | 16 (18%) | 48 |  | 27 |  |
| TRAIL | 3 (3%) | 30 |  | 64 |  | 5 (5%) | 35 |  | 51 |  |
| SDF-1a+b | 35 (36%) | 41 |  | 21 |  | 32 (35%) | 35 |  | 24 |  |
| ENA-78 | 91 (94%) | 1 |  | 5 |  | 85 (93%) | 4 |  | 2 |  |
| MIP1-d | 6 (6%) | 60 |  | 29 | 2 | 8 (9%) | 59 |  | 23 | 1 |
| IL-28A | 3 (3%) | 82 |  | 12 |  | 4 (4%) | 78 |  | 9 |  |

Values are shown as number (%).

OOR< = fluorescence under the detection limit; OOR> = fluorescence exceeds of detection limit; * = sample value extrapolated; *** = sample missing

Cytokine concentrations measured by multiplex ELISA using fluorescence from PBMC culture medium

*Detailed version of the PBMC extraction protocol*

Blood samples for peripheral blood mononuclear cell (PBMC) processing (>8 ml) were collected to LH Lithium Heparin tube in two different time points (At the study entry and at the convalescent phase (2-week follow-up)). Both samples were analyzed with identical fashion and using the same procedure. After the blood sample was collected, the samples were placed in a rocking shaker and were slowly rocked in a room temperature until the beginning of PBMC processing, which was performed on the same day.

At the beginning of PBMC processing, the sample was centrifuged at 1200 rpm for 10 minutes in +22°C. Thereafter, the plasma was extracted to 15ml falcon tube and frozen. After the plasma extraction, the remaining cell pellet was moved to 50 ml falcon tube after which a phosphate-buffered saline (PBS) solution was added on the top of the cell pellet (final volume of the mixture 20 ml). The sample-PBS-mixture was then carefully divided into two new 15ml falcon tubes each containing 3 ml of room temperature Ficoll-Plaque™ PLUS (GE Healthcare, Amersham, United Kingdom). The mixture was then centrifuged at 2200 rpm for 22 minutes in +22°C. All the visible PBS was then extracted with pipette and PBMCs from both 15 ml falcon tubes was extracted with Pasteur pipette to a new 50 ml falcon tube. 30 ml of PBS was then added on the top of the PBMCs, and the mixture then centrifuged at 1200 rpm for 10 minutes in +22°C. All visible PBS was then extracted, and solution was diluted with 1 ml of PBS. 10 µl of PBMC-PBS-mixture and 90 µl of TryptanBlue (0.5%) was then mixed in Eppendorf tube, and the cells were placed in a Bürker chamber, and the cells were calculated.

After cell calculation, mixture was then diluted that the final sample would contain 2 million cells per ml. Adequately diluted sample was then carefully divided to a 48-well culture plate (one stimulant per well) containing the stimulants (Supplementary Table 8). Each well contained 0.5x10^6 PBMCs (in volume of 250 µl) and stimulant (25 µl) diluted to 225 µl of medium (total volume was 500 µl/well). PBMCs were stimulated with anti-CD3/anti-CD28 ([20 µg/ml / 20 µg/ml; final 1 µg/ml / 1 µg/ml; i.e. 0.5 µg/well] BD Biosciences, Franklin Lakes, NJ, USA) in 5% CO2 incubator for 24h and after that the cell and the medium mixture were collected to 2 ml Eppendorf tubes. The samples were centrifuged in 1200 rpm, 1 min at room temperature after which the supernatants were collected and stored in a -80°C refrigerator.

Later, the supernatants were shipped inside dry ice containers to the Swiss Institute of Allergy and Asthma Research (SIAF), Davos, Switzerland. Upon arrival the samples were still frozen and stored in -80°C refrigerator until analysis. Samples were defrosted right before the analyses and analyzed with Millipore HCYTOMAG-60K-36 and HCYP2MAG-62K-20 assay (Merck KGaA, Darmstadt, Germany) using the Bio-Plex 200 System utilizing the Bio-Plex Manager 6.0 Software (Bio-Rad, Cressier, Switzerland) to perform profiling of 56 different cytokines (Supplementary Table 1-3). Internal quality controls for all analytes were satisfactory.

*Stimulants*

More than 700 vials of anti-CD3/anti-CD28 20μg/ml /20μg/ml (final 1 μg/ml / 1 μg/ml), i.e. 0.5 μg/well (BD Biosciences, Franklin Lakes, NJ, USA) were frozen before study (300 to be used at acute phase, 300 at convalescent phase and 100 for reserve). The stimulants were prepared prior to the study at the same time to ensure that concentration of the stimulants remained stable throughout the study period. Each stimulant was be frozen in 25 μl aliquots in –80 °C and defrosted in room temperature just before use in PBMC cultures (0.5 x 10^6^ cells/well, final volume 0.5 ml). All stimulants and their incubation times are shown in Supplementary Table 8.

*Medium*

Medium contained 100 ml RPMI-16400, 10 ml iFBS, 1 ml L-glutamine, 20 µl gentamicin, 2 ml HEPES.

*Cytokine analyses*

Due to the limitations of quantitative multiplex Elisa profiling, a few cytokines did not reach the quantitative limit of detection (i.e., fluorescence was under or exceeded the quantification limit of the assay) (Supplementary Table S3). These values were identified as lower limit of quantification (LLOQ) and upper limit of quantification (ULOQ). Each cytokine found in more than 50% of patient samples within the limit of quantification (values between LLOQ and ULOQ) were included for analysis, thus ensuring that conclusions would not be based on minority of samples (Supplementary Table S1-2). Sample was classified as out of range when signal, i.e., fluorescence fell out of the assay range which was both precise and accurate. Due to aforementioned, of 56 cytokines 29 were later classified as eligible for statistical analyses. Samples under the limit of detection were assigned half the value of the LLOQ (Supplementary Table 1) (1,2), and samples exceeding the upper limit of detection (either reported as “ULOQ” or a numerical extrapolated value greater than the assays upper limit of quantification), the values were set to the ULOQ threshold of the assay (Supplementary Table 2) (3).

References for statistics:

1. Sehmi R., Lim H. F., Mukherjee M., Huang,C., Radford K., Newbold P., et al. Benralizumab attenuates airway eosinophilia in prednisone-dependent asthma. *J Allergy Clin Immunol* (2018) 141:1529-1532.e8. doi: 10.1016/J.JACI.2018.01.008

2. Kolbinger F., Loesche C., Valentin,M. A., Jiang X., Cheng Y., Jarvis P., et al. β-Defensin 2 is a responsive biomarker of IL-17A-driven skin pathology in patients with psoriasis. *J Allergy Clin Immunol* (2017) 139:923-932.e8. doi: 10.1016/J.JACI.2016.06.038

3. Laetsch T.W., Myers G.D., Baruchel A., Dietz A.C., Pulsipher M.A., Bittencour, H., et al. Patient-reported quality of life after tisagenlecleucel infusion in children and young adults with relapsed or refractory B-cell acute lymphoblastic leukaemia: a global, single-arm, phase 2 trial. *Lancet Oncol* (2019) 20:1710–1718. doi: 10.1016/S1470-2045(19)30493-0

*Detailed version of the statistics*

Differences in the baseline characteristics between the groups were analyzed by using two-sample t-test for normally distributed and Mann-Whitney U-test for non-normally distributed data. The normality of distribution was assessed by Kolmogorov-Smirnov test. Categorial variables were analyzed using χ^2^ test or Fisher exact test (when cell counts < 5). Due to positively and negatively skewed distribution, continuous cytokine variables data were normalized using log or x² transformation before regression analyses.

Differences between study groups in cytokine expression were first analyzed using Mann-Whitney U test for non-normally distributed data and secondly after log- or x²-transformation using multivariable linear model analysis. The adjustments for analyses included baseline characteristics which significantly differed between the groups (age, weight, duration of previous symptoms [rhinitis, cough, wheezing, fever] and B-Eos at entry). Backward stepwise method was used for the final adjustment model separately for each cytokine. Only statistically significant variables (*P* < .05) were included in the final model.

Differences between the RV-serotypes in the cytokine expression were analyzed using Kruskal–Wallis H test for non-normally distributed data. Negative binominal regression was used to analyze the effects of viral group (RV vs RSV) and cytokine expression on duration of hospitalization. Group × cytokine level interaction effect was included in models and if a statistically significant interaction was found, the cytokine level effect was estimated separately in the RV and the RSV groups. If the interaction was not statistically significant, the effects for the group and the cytokine level were estimated from the main effects model.

The association between the cytokine expression and the time to a new physician-confirmed wheezing episode within 2 and 12 months was analyzed with Mann Whiney U-test. However, due to the scarcity of events in the RSV group, only the RV group was included in the analyses. A two-sided P value < .05 was considered statistically significant. Data analyses were made using JMP software (version 13.1.0, SAS Institute, Cary, NC, USA).

**Supplementary Table 4**: Differences in cytokine expression levels at study entry, and the convalescent phase

| Cytokine | Timing | RV  (acute)=47  n(convalescent)=20  n(difference)=17 | RSV  n(acute)=9  n(convalescent)=10  n(difference)=8 | p-value, univariate | p-value, multivariate | Adjustments |
| --- | --- | --- | --- | --- | --- | --- |
| EGF | Acute  Convalescent  Difference | 3.50 (1.6–4.3)  -  - | 4.0 (2.5–6.5)  -  - | .32  -  - | *.38*  *-*  *-* | -  *-*  *-* |
| G-CSF | Acute  Convalescent  Difference | 10 (7.9–32)  10 (8.9–640)  3.1 (-4.1–570) | 15 (6.5–460)  8.9 (8.7–140)  -1.4 (-520–84) | .30  .35  .26 | *.23*  *.42*  *.28* | *1,6*  -  *1,2* |
| GM-CSF | Acute  Convalescent  Difference | 12 (1.6–110)  -  - | 36 (1.6–310)  -  - | .65  -  - | *.65*  *-*  *-* | -  *-*  *-* |
| Fractalkine | Acute  Convalescent  Difference | 14 (8.3–28)  15 (9.3–21)  1.1 (-6.4–8.2) | 13 (8.3–24)  8.3 (7.4–13)  -4.6 (-17–8.4) | .46  **.03**  .35 | *.73*  ***.02***  ***.03*** | *4*  -  *1,3* |
| IFNa2 | Acute  Convalescent  Difference | 5.1 (3.0–68)  2.1 (1.5–5.0)  -2.1 (-34–2.2) | 5.4 (2.1–64)  6.3 (1.5–82)  2.0 (-4.5–11) | .66  .23  .17 | *.53*  *.12*  *.40* | *3,4*  - |
| IFNg | Acute  Convalescent  Difference | 9.5 (1.5–81)  12 (1.6–390)  0.15 (-11–260) | 13 (1.6–180)  12 (3.0–150)  -7.8 (-160–18) | .56  .93  .27 | *.30*  *.81*  *.50* | *1*  -  - |
| IL-10 | Acute  Convalescent  Difference | 37 (5.3–130)  52 (7.1–380)  1.4 (-190–290) | 67 (3.5–540)  84 (7.3–160)  -33 (-660–120) | .70  .79  .38 | *.21*  *.55*  *.38* | *1*  *1,2*  - |
| MCP-3 | Acute  Convalescent  Difference | 300 (120–390)  390 (36–390)  92 (-98–340) | 200 (66–570)  370 (47–390)  -62 (-380–160) | .96  .91  .16 | *.92*  *.29*  *.92* | -  *4*  - |
| MDC | Acute  Convalescent  Difference | 250 (130–1400)  290 (150–1400)  -26 (-1400–680) | 370 (67–3400)  470 (74–1900)  160 (-4200–2100) | .87  .96  .73 | *.69*  *.84*  *.92* | *5,6*  -  - |
| IL-13 | Acute  Convalescent  Difference | 5.5 (1.5–20)  3.2 (1.5–13)  0 (-9.0–9.8) | 4.8 (1.5–18)  7.2 (2.7–62)  2.6 (-15–42) | .60  .24  .77 | *.44*  *.72*  *.56* | -  *3,4,7*  - |
| IL-1RA | Acute  Convalescent  Difference | 97 (28–420)  170 (50–340)  24 (-280–200) | 240 (78–600)  140 (15–320)  -56 (-560–280) | .23  .57  .82 | *.****048***  *.98*  *.36* | *1*  *3*  - |
| IL-1b | Acute  Convalescent  Difference | 3.5 (1.6–25)  25 (1.6–270)  5.0 (0–220) | 30 (1.6–1400)  4.2 (1.6–120)  -6.8 (-1600–24) | .13  .32  .13 | ***.008***  *.27*  ***.03*** | *1*  -  - |
| IL-2 | Acute  Convalescent  Difference | 3.4 (1.6–21)  3.6 (1.6–21)  0 (-7.7–11) | 3.2 (1.6–15)  3.2 (1.6–69)  0 (-8.7–41) | .68  .98  .86 | *.52*  *.82*  *.99* | -  -  - |
| IL-6 | Acute  Convalescent  Difference | 32 (6.8–380)  66 (4.2–2000)  2.4 (-31–2000) | 110 (17–2100)  29 (9.5– 960)  -61 (-2100–28) | .28  .68  .10 | *.056*  *.70*  *.06* | *1*  -  - |
| IL-8 | Acute  Convalescent  Difference | 1400 (650–2500)  840 (600–1600)  -10 (-1300–960) | 1200 (590–3500)  2000 (550–3800)  -600 (-1600–750) | .81  .63  .35 | *.30*  *.52*  *.90* | *5*  -  *1,2* |
| IP-10 | Acute  Convalescent  Difference | 1200 (340–10000)  640 (170–3000)  -380 (-8000–430) | 1300 (120–6800)  950 (400–10000)  220 (-8200–9500) | .80  .39  .32 | *.76*  *.37*  *.33* | -  -  - |
| MCP-1 | Acute  Convalescent  Difference | 6900 (4200–8300)  6700 (4300–8300)  0 (-3800–2700) | 7500 (5400–8300)  6700 (4900–7700)  -160 (-2200–1100) | .60  .93  .68 | *.****04***  *.09*  *.55* | *1*  *1,2,5*  *1,2* |
| MIP-1a | Acute  Convalescent  Difference | 64 (10–440)  180 (3.5–550)  2.2 (-29–430) | 84 (8.7–1600)  150 (4.9–390)  -36 (-1400–28) | .58  .89  .10 | *.54*  *.91*  *.15* | -  -  - |
| MIP-1b | Acute  Convalescent  Difference | 210 (58–940)  430 (48–1300)  150 (-300–640) | 170 (67–1700)  290 (32–960)  -100 (-1400–220) | .76  .60  .20 | *.77*  *.69*  *.14* | -  -  - |
| RANTES | Acute  Convalescent  Difference | 300 (120–680)  250 (69–800)  -130 (-320–300) | 230 (210–340)  510 (94–970)  320 (-170–730) | .44  .54  .35 | *.28*  *.28*  *.82* | *5*  *4*  - |
| TNFa | Acute  Convalescent  Difference | 65 (25–1100)  420 (11–1300)  140 (-50–1100) | 200 (29–1800)  140 (32–1000)  -130 (-1100–270) | .37  .63  .081 | *.11*  *.92*  *.19* | *1*  -  - |
| VEGF | Acute  Convalescent  Difference | 63 (12–210)  -  - | 30 (14–140)  -  - | .45  -  - | *.84*  *-*  *-* | *4*  *-*  *-* |
| Eotaxin-2 | Acute  Convalescent  Difference | 740 (640–980)  770 (450–1100)  -52 (–340–320) | 350 (180–740)  520 (330–980)  37 (-140–290) | **.01**  .25  .56 | ***.01***  *.81*  *.36* | -  *1,2,5*  *1,2* |
| MCP-2 | Acute  Convalescent  Difference | 610 (200–1200)  230 (120–560)  -360 (-770–(-70)) | 120 (68–970)  150 (49–220)  -47 (-390–120) | .18  .25  .15 | *.69*  *.14*  *.47* | *2*  *6*  *1,2* |
| I-309 | Acute  Convalescent  Difference | 32 (19–67)  24 (14–41)  -8.4 (-20–7.8) | 12 (7.8–21)  29 (11–34)  10 (3.8–17) | **.001**  .68  **.02** | *.08*  *.58*  ***.046*** | 2  *5*  - |
| IL-16 | Acute  Convalescent  Difference | 66 (50–90)  47 (34–73)  -14 (–49–4.7) | 76 (53–93)  67 (44–83)  -7.1 (–34–48) | .76  .42  .50 | *.17*  *.22*  *.29* | *2*  -  - |
| TARC | Acute  Convalescent  Difference | 3.9 (2.0–8.9)  4.4 (2.4–7.8)  -0.96 (-2.4–2.5) | 1.2 (0.88–2.1)  2.9 (1.9–3.6)  1.4 (0.28–1.9) | **.002**  .31  .11 | ***.002***  *.64*  ***.03*** | -  *6,7*  - |
| Eotaxin-3 | Acute  Convalescent  Difference | 110 (110–220)  -  - | 110 (110–160)  -  - | .28  -  - | *.07*  *-*  *-* | *7*  *-*  *-* |
| ENA-78 | Acute  Convalescent  Difference | 900 (170–2200)  190 (65–1800)  -160 (-890–68) | 210 (73–470)  230 (50–1100)  -30 (-200–440) | **.02**  .86  .20 | ***.03***  *.65*  *.32* | -  *1,2,5*  *2* |

Acute sample, samples drawn at the study entry; Convalescent sample, samples drawn at the 2-week follow-up; Difference, difference in cytokine expression when comparing samples drawn at the 2-week follow-up and at the study entry.

Values are shown as medians (interquartile range).

Data were analyzed by Mann-Whitney U-test, and analysis, and by multivariable linear model analysis (after log- or x²-transformation). The adjustments for immunologic analyses included baseline characteristics that significantly differed between the groups (Age = 1, weight = 2, duration of previous symptoms (rhinitis = 3, cough = 4, wheezing =5, fever = 6), and B-Eos = 7 at entry). A backward stepwise method was used for the final adjustment model separately for each cytokine. Only statistically significant variables (*P* < .05) were included in the final model.

**Supplementary Table 5**: The biological mechanisms of significant cytokines

| Abbrevation | Cytokine | Biological mechanism | Expression in respiratory virus infection, asthma, and allergy |
| --- | --- | --- | --- |
| Fractalkine | CX3CL1 | Attraction of monocytes, activated T cells, NK cells, and microglia cells (1,2). | Upregulation in lungs after RSV infection (3). Cell culture and mouse studies suggest possible RSV G protein interference through CX3CR1 blocking (4). Patients with fungal-associated allergic asthma express higher levels of fractalkine and it is suggested to have protective role in this situation (5)*.* |
| IL-1b | Interleukin 1 beta | Induction of proinflammatory proteins, differentiation of Th17 cells (6). | Increased expression and role in immunopathology in RSV infection (7-9) and activation in rhinovirus infections (10). Potentially regulative effect on the lung immune response or tissue injury caused by RSV (11). Promotes inflammation in patients with asthma and persistent allergic rhinitis (11,12). Mouse studies suggest that inhibition of IL-1b could decrease RSV immunopathology and this way possibly asthma exacerbation (13). |
| Eotaxin-2 | Eosinophil chemotactic protein 2, CCL24 | Chemotaxis of eosinophils, basophils, Th2 cells, mast cells, and subsets of immature dendritic cells (14-16). | Exposure to RV increased the protein levels of eotaxin-2 (17), although smaller increase in children with asthma compared to children without asthma (18), indicating that infection significantly amplifies the underlying type 2 response. Inhibition of CCL24 reduces disease severity in a model of RV-induced asthma exacerbation (19). RV infection up-regulates eotaxin-2 expression in bronchial epithelial cells (20). In the absence of viral infection, higher eotaxin-2 levels are associated with decreasing lung function. However, with infection, cytokine abundance is associated with reduced lung function decline (21). |
| I-309 | CCL1 | Antimicrobial gene, attracts monocytes and activated Th2 cells (22,23). | RSV induced increase in I-309 expression in basal epithelial cells (3). Upregulation of I-309 was also detected in broncho alveolar fluids from asthmatics compared to normal (24,25). An acute exacerbation was not detected to increase I-309 expression in asthmatic patients in contrast to patients with stable asthma (26). |
| TARC | Thymus- and activation-regulated chemokine, CCL17 | Attraction of new TARC producing mature dendritic cells and Th2 cells (27). | Both RV (28,29) and RSV (30) have been detected to increase TARC production measured from serum and nasal fluids. Mouse studies and studies with asthmatic patients have shown significantly higher increase in TARC excretion in asthma patients or previously allergen sensitized mice infected with RV compared to non-asthmatic and non-sensitized controls (28,31). Higher production of TARC in lungs has been seen in mouse studies (32). |
| ENA-78 | Epithelial-derived neutrophil-activating peptide 78, CXCL5 | Neutrophil attraction and activation via eosinophils, angiogenic properties (33). | Human airway epithelial and peripheral blood mononuclear cells affected with RV has shown significantly higher ENA-78 levels, than uninfected controls, and associated with RV infection (34,35). RV infection induced significantly higher ENA-78 expression in asthmatic patients compared to non-asthmatic controls (36). |
| IL-1RA | Interleukin 1 receptor antagonist | Inhibits the proinflammatory effects of IL-1b and IL-1a (37). | Increased expression of IL-1RA in both RV (38) and RSV (39,40) infections has been shown in nasopharyngeal samples and in vitro studies. Nasal IL-1RA levels were higher in non-asthmatics when compared with asthmatic patients during RV infection (41). Fungal-associated asthmatics had elevated IL-1RA levels in lung lavage fluid compared to healthy controls (42). Generally, in asthmatics higher levels of serum IL-1RA was associated with lower subsequent risks of worsening asthma (43). |
| IL-6 | Interleukin 6 | Stimulation of acute phase protein synthesis, development of neutrophils and B cells. Essential for development of Th22 and Th17 (44-46). | In cell studies both RV (47) and RSV (48) infection has been connected to higher levels of IL-6 compared to non-infected controls. In patients with acute phase RSV infection IL-6 increased significantly and higher IL-6 levels were associated with more severe disease (49). Compared to stable asthma and healthy controls the patients with acute asthma attacks had elevated IL-6 levels in sputum (50). |
| GM-CSF | Granulocyte-macrophage colony-stimulating factor | Production of neutrophils, eosinophils, basophils, and monocytes (51,52). | RV infection induced higher GM-CSF levels have been observed in studies with human bronchial epithelial cells (53,54). The GM-CSF concentration level in sputum has been detected higher in asthmatics, with moderate and severe disease compared to healthy controls, and correlated with eosinophil count (55). In other study lower peripheral blood GM-CSF was associated with acute asthma exacerbation (56), but some studies did not detect significantly changed levels in asthmatics or in RV infection (57,58). In mouse studies post-allergic airway inflammation lungs had reduced levels of GM-CSF (59). With RSV infected epithelial cells GM-CSF release significantly enhanced (60). |
| IFN-g | Interferon gamma | Attraction of Th1 cells and monocytes via IP-10 and MIG (61). | The nasal levels of IFNg increased during RV infection in both controls and children with asthma (18,62). In patients with no allergies RV infection upregulated IFN-g expression, but with allergic patients there was no significant increase detected (63). With RSV infection IFN-g production has been shown to decrease (64), and in children, weaker IFN-g response have been associated with RSV susceptibility (65). |
| MDC | Macrophage-derived chemokine, CCL22 | Monocyte, dendritic cell, NK cell and activated Th2 cell attraction (66). | RV infection induced MDC expression in nasal samples, in vitro epithelial cells from donors with asthma and in vivo mice studies. MDC levels did not differ significantly between asthmatics and healthy controls, but in RV infection MDC levels were significantly higher for asthmatics (29,67). RSV infected lower epithelial cells have been detected with higher expression of MDC (3). RSV-specific memory CD8 T cells have been shown to inhibit the production of MDC (68). |
| IL-13 | Interleukin 13 | Activation of monocyte cell lines and inhibition of inflammatory cytokines such as IL-1a, IL-1b, IL-6, IL-8, G-CSF and IFNa. Secreted by Th2 cells (69,70). | Mouse studies have shown higher bronchoalveolar levels of IL-13 with RV infected mice (71,72), but other studies have detected significantly increased IL-13 expression in RV infection only with patients with asthma (62) and mice sensitized with allergen (31) compared to healthy and not-sensitized controls. Primary bronchial epithelial cells from asthmatic and wheezing children had increased secretion of IL-13 in response to RSV compared to controls (73). Higher sputum IL-13 from infants with severe RSV bronchiolitis has been associated with risk of developing recurrent wheezing (74). |
| MIP-1b | Macrophage inflammatory protein 1 beta, CCL4 | Chemoattraction for eosinophils, monocytes, B cells, and immature dendritic cells. Macrophage activation (75). | In vivo mouse studies and in vitro cell studies have shown induced expression of MDC in RSV infection (76-79). Also, patients hospitalized because of RSV infection have shown increased MDC mRNA expression (80). Children with asthma had statistically higher concentrations of MIP-1b during RSV and other respiratory infections compared to non-asthmatic controls (21,40,81). |
| VEGF | Vascular endothelial growth factor | Vascular angiogenesis, remodeling, inducer of eosinophilic inflammation, mucus production, chemotactic for monocytes, Th2 inflammation (82). | RSV infection has been connected to increased acute (39,83) and persistent long-term (84) expression of VEGF in patients with RSV infection and in vitro cell studies. Enhanced VEGF expression has also been detected in RV infected cells in vivo and in vitro studies (85,86). Asthma patients had higher levels of sputum VEGF compared to healthy controls and the VEGF expression significantly increased in acute asthma compared to remission (87). Single nucleotide polymorphisms of VEGF were associated with risk of developing wheezing or recurrent wheezing in infants with first acute wheezing episode (88). |
| MCP-1 | Monocyte chemoattractant protein 1, CCL2 | Attraction of basophils, monocytes, activated T cells, NK cells, and immature dendritic cells (89). | In vitro models of peripheral blood mononuclear cells and bronchoalveolar lavage macrophages infected with RV induced secretion of MCP-1 (90). |

**Supplementary Table 6**: Association between cytokine expression and severity of acute illness.

| Outcome  Duration of hospitalization  Log-transformed cytokine level |  |  |  |  |  |
| --- | --- | --- | --- | --- | --- |
|  | Group effect  RV vs RSV    Estimate  (95% CI) *P* | | Cytokine effect  Expression of cytokine  Estimate  (95% CI) *P* | | Group × cytokine interaction effect  *P* |
|  |  |  |  |  |  |
| EGF | 0.706^‡^ (0.491, 1.016) | .06 | 0.996^¶^ (0.587, 1.689) | .99 | .41 |
| G-CSF | 0.698^‡^ (0.485, 1.003) | .05 | 0.929^¶^ (0.748, 1.153) | .50 | .23 |
| GM-CSF | 0.677^‡^ (0.461, 0.996) | .048 | 0.946^¶^ (0.807, 1.109) | .50 | .28 |
| Fractalkine | 0.770^‡^ (0.521, 1.136) | .11 | 0.770^¶^ (0.521, 1.114) | .19 | .13 |
| IFNa2 | 0.707^‡^ (0.491, 1.016) | .06 | 0.989^¶^ (0.823, 1.189) | .90 | .44 |
| IFN-g |  |  | 1.033^*^ (0.878, 1.216)  0.651^†^ (0.460, 0.922) | .69^§^  .**02**^#^ | .**03** |
| IL-10 | 0.714^‡^ (0.497, 1.026) | .07 | 0.942^¶^ (0.798, 1.112) | .48 | .07 |
| MCP-3 | 0.696^‡^ (0.483, 1.005) | .05 | 1.089^¶^ (0.794, 1.490) | .60 | .15 |
| MDC |  |  | 1.047^*^ (0.841, 1.304)  0.609^†^ (0.428, 0.868) | .68^§^  .**001**^#^ | .**02** |
| IL-13 | 0.690^‡^ (0.477, 0.999) | .049 | 1.058^¶^ (0.880, 1.271) | .55 | .39 |
| IL-1RA |  |  | 1.026^*^ (0.825, 1.276)  0.481^†^ (0.289, 0.801) | .82^§^  .**005**^#^ | .**02** |
| IL-1b | 0.703^‡^ (0.487, 1.014) | .06 | 0.986^¶^ (0.874, 1.148) | .86 | .13 |
| IL-2 | 0.699^‡^ (0.483, 1.012) | .06 | 1.032^¶^ (0.823, 1.285) | .78 | .26 |
| IL-6 |  |  | 1.088^*^ (0.938, 1.262)  0.764^†^ (0.566, 1.032) | .26^§^  .08^#^ | .**04** |
| IL-8 | 0.716^‡^ (0.496, 1.033) | .07 | 0.930^¶^ (0.670, 1.292) | .67 | .08 |
| IP-10 | 0.700^‡^ (0.486, 1.007) | .06 | 1.043^¶^ (0.874, 1.244) | .64 | .36 |
| MCP-1 | 0.700^‡^ (0.489, 1.002) | .05 | 1.583^¶^ (0.748, 3.350) | .23 | .24 |
| MIP-1a | 0.705^‡^ (0.491, 1.012) | .06 | 1.042^¶^ (0.900, 1.205) | .58 | .11 |
| MIP-1b | 0.696^‡^ (0.485, 0.999) | .049 | 1.106^¶^ (0.895, 1.367) | .35 | .10 |
| RANTES | 0.704^‡^ (0.491, 1.008) | .06 | 1.142^¶^ (0.873, 1.493) | .33 | .46 |
| TNFa | 0.710^‡^ (0.494, 1.021) | .07 | 1.052^¶^ (0.890, 1.242) | .55 | .07 |
| VEGF |  |  | 1.190^*^ (0.940, 1.510)  0.385^†^ (0.290, 0.530) | .16^§^  <.**0001**^#^ | .**0004** |
| Eotaxin-2 | 0.835^‡^ (0.554, 1.259) | .39 | 0.729^¶^ (0.487, 1.090) | .12 | .30 |
| MCP-2 | 0.696^‡^ (0.485, 0.999) | .049 | 1.100^¶^ (0.893, 1.354) | .37 | .13 |
| I-309 | 0.745^‡^ (0.505, 1.098) | .14 | 0.874^¶^ (0.613, 1.247) | .46 | .13 |
| IL-16 | 0.726^‡^ (0.504, 1.046) | .09 | 1.380^¶^ (0.643, 2.964) | .41 | .15 |
| TARC | 0.753^‡^ (0.507, 1.117) | .16 | 0.871^¶^ (0.615, 1.233) | .43 | .33 |
| Eotaxin-3 | 0.720^‡^ (0.499, 1.041) | .08 | 0.823^¶^ (0.431, 1.573) | .56 | .87 |
| ENA-78 | 0.766^‡^ (0.507, 1.157) | .21 | 0.913^¶^ (0.729, 1.144) | .43 | .26 |

CI = confidence interval

^*^ Relative risk; RV-group negative binomial regression

^†^ Relative risk; RSV-group negative binomial regression

^¶^ Relative risk

^‡^ Binary logistic regression

^§^ Group effect in RV treatment arm

^#^ Group effect in placebo treatment arm

Due to the significant interactions, the cytokine effect was not estimated using all data. The effect of cytokine is presented separately in the RV and RSV groups.

**Supplementary Table 7**: The association between cytokine expression and recurrencies

|  |  | 2 months | | | 12 months | | |
| --- | --- | --- | --- | --- | --- | --- | --- |
| Cytokine | Timing | No relapse  n(acute) = 10  n(convalescent) = 10  n(difference) = 9 | Relapse  n(acute) = 11  n(convalescent) = 10  n(difference) = 8 | p-value | No relapse  n(acute) = 4  n(convalescent) = 4  n(difference) = 3 | Relapse  n(acute) = 17  n(convalescent) = 16  n(difference) = 14 | p-value |
| EGF | Acute samples  Convalescent samples  Difference | 4.2 (2.8–4.4)  -  - | 3.5 (1.6–4.1)  -  - | .17  -  - | 4.2 (2.2–4.6)  -  - | 3.5 (1.6–4.2)  -  - | .36  -  - |
| G-CSF | Acute samples  Convalescent samples  Difference | 9.6 (7.3–590)  25 (7.6–800)  6.2 (-240–900) | 8.8 (4.1–32)  9.0 (8.9–930)  1.5 (0–13) | .52  .76  .77 | 9.6 (7.6–610)  570 (120–1850)  700 (430–2200) | 8.9 (5.8–230)  9.7 (8.9–200)  1.4 (-51–8.5) | .65  .22  **.04** |
| GM-CSF | Acute samples  Convalescent samples  Difference | 4.2 (1.6–270)  -  - | 8.3 (1.6–290)  -  - | .61  -  - | 1.6 (1.6–1.6)  -  - | 8.3 (1.6–230)  -  - | .10  -  - |
| Fractalkine | Acute samples  Convalescent samples  Difference | 14 (8.3–30)  16 (8.0–24)  0 (-6.4–8.7) | 14 (8.3–28)  13 (9.3–20)  3.0 (-12–79) | .89  .73  .81 | 9.5 (5.9–25)  570 (120–1850)  9.3 (8.1–16) | 17 (8.3–28)  13 (8.5–20)  0 (-9.7–5.4) | .28  .07  **.02** |
| IFNa2 | Acute samples  Convalescent samples  Difference | 4.3 (2.6–20)  1.5 (1.5–14)  0 (-2.9–15) | 6.0 (3.5–68)  2.7 (1.5–4.9)  -22 (-71– (-2.3)) | .27  .81  **.02** | 3.8 (1.8–13)  5.9 (1.5–22)  5.7 (-1.5–24) | 5.4 (3.4–55)  2.1 (1.5–4.8)  -3.6 (-44–0.21) | .23  .69  .06 |
| IFNg | Acute samples  Convalescent samples  Difference | 9.3 (1.5–51)  63 (1.6–400)  3.7 (-11–320) | 14 (1.5–81)  8.0 (1.6–410)  0.07 (-12–160) | .91  .54  .60 | 1.5 (1.5–100)  320 (32–1400)  110 (3.7–1700) | 14 (1.5–53)  8.0 (1.6–350)  0.07 (-13–230) | .39  .07  .13 |
| IL-10 | Acute samples  Convalescent samples  Difference | 8.3 (4.9–480)  60 (7.9–540)  12 (-270–590) | 82 (6.0–340)  52 (5.4–300)  2.7 (-250–90) | .57  .79  .63 | 4.6 (2.2–340)  290 (35–960)  470 (12–1100) | 20 (5.7–350)  35 (6.3–110)  -2.4 (-350–99) | .13  .19  .10 |
| MCP-3 | Acute samples  Convalescent samples  Difference | 240 (64–390)  390 (150–510)  270 (-120–500) | 220 (82–390)  250 (21–390)  27 (-110–270) | .89  .48  .23 | 91 (21–790)  390 (270–390)  330 (270–380) | 250 (110–390)  290 (18–390)  27 (-150–340) | .28  .46  .13 |
| MDC | Acute samples  Convalescent samples  Difference | 280 (220–2100)  230 (96–1900)  -26 (-2000–1800) | 600 (160–1700)  1000 (240–1600)  -120 (-1000–700) | .89  .41  .92 | 210 (130–290)  1700 (150–6200)  2900 (-26–7100) | 650 (240–2200)  290 (130–1300)  -290 (-1800–620) | .11  .45  .06 |
| IL-13 | Acute samples  Convalescent samples  Difference | 5.3 (1.5–16)  4.7 (1.5–24)  0 (-15–9.8) | 5.0 (1.5–9.4)  3.2 (1.5–33)  0.3 (-3.4–83) | .97  .85  .66 | 1.5 (1.5–2.6)  12 (3.5–42)  8.3 (0–11) | 6.0 (3.6–17)  3.0 (1.5–7.6)  -1.4 (-14–29) | **.02**  .31  .28 |
| IL-1RA | Acute samples  Convalescent samples  Difference | 130 (30–320)  140 (33–290)  78 (-280–220) | 100 (75–300)  200 (58–390)  19 (-310–190) | .67  .45  .56 | 47 (15–180)  210 (43–550)  280 (78–590) | 110 (80–400)  170 (50–330)  -0.02 (-310–120) | .13  .71  **.03** |
| IL-1b | Acute samples  Convalescent samples  Difference | 1.6 (1.6–1100)  63 (1.6–2000)  12 (-500–3700) | 1.6 (1.6–25)  20 (1.6–500)  2.5 (0–30) | .74  .51  .56 | 1.6 (1.6–2300)  220 (40–6300)  330 (110–8300) | 1.6 (1.6–170)  12 (1.6–92)  0 (-250–28) | .84  .09  **.03** |
| IL-2 | Acute samples  Convalescent samples  Difference | 1.6 (1.6–6.4)  3.4 (1.6–20)  0 (-7.7–11) | 1.6 (1.6–41)  3.6 (1.6–43)  0 (-15–74) | .38  .63  .85 | 1.6 (1.6–1.6)  14 (2.5–25)  3.8 (0–20) | 1.6 (1.6–31)  2.4 (1.6–16)  0 (-15–7.3) | .10  .32  .25 |
| IL-6 | Acute samples  Convalescent samples  Difference | 13 (3.7–2000)  300 (3.4–2000)  2.4 (-700–2000) | 55 (5.4–280)  66 (4.1–2400)  4.5 (-37–100) | .89  .82  .92 | 7.8 (2.3–1500)  2000 (510–7200)  2000 (2000–9000) | 55 (5.9–1100)  54 (2.2–1300)  -2.7 (-150–76) | .42  .09  **.02** |
| IL-8 | Acute samples  Convalescent samples  Difference | 1100 (470–2700)  780 (620–1500)  270 (-1700–960) | 2000 (780–2600)  960 (540–4500)  -190 (-1200–1000) | .32  .65  .92 | 600 (200–3600)  1100 (720–6700)  1300 (420–3900) | 2000 (660–2600)  840 (450–1600)  -270 (-1500–350) | .24  .51  .06 |
| IP-10 | Acute samples  Convalescent samples  Difference | 730 (370–8100)  810 (120–2500)  -380 (-8000–1100) | 3700 (120–10000)  760 (230–3900)  -1100 (-8500–120) | .72  .88  .77 | 540 (170–800)  3000 (610–8500)  2000 (-380–9500) | 3700 (340–10000)  580 (140–1700)  -1200 (-9300–140) | .15  .16  .08 |
| MCP-1 | Acute samples  Convalescent samples  Difference | 6400 (3900–8300)  4700 (2500–8300)  0 (-5100–3000) | 7500 (4900–8300)  7900 (6200–8300)  0 (-500–2500) | .47  .09  .85 | 4200 (2200–7500)  6500 (4700–8300)  3300 (2700–5100) | 7500 (5300–8300)  6700 (3200–8300)  -150 (-5000–1500) | .11  .70  **.02** |
| MIP-1a | Acute samples  Convalescent samples  Difference | 38 (4.9–680)  180 (2.9–480)  2.2 (-400–290) | 42 (4.3–1500)  250 (3.7–1700)  31 (-39–1000) | .80  .47  .70 | 66 (2.6–360)  180 (37–360)  85 (-4.4–140) | 42 (7.4–1400)  250 (3.5–1000)  1.1 (-240–600) | .56  .54  .70 |
| MIP-1b | Acute samples  Convalescent samples  Difference | 70 (27–900)  430 (48–1100)  380 (-410–1100) | 210 (120–1400)  450 (41–1400)  26 (-370–580) | .12  .94  .50 | 27 (19–1000)  430 (390–1920)  420 (380–2400) | 200 (70–1200)  430 (32–1300)  -25 (-500–620) | .06  .45  .10 |
| RANTES | Acute samples  Convalescent samples  Difference | 250 (46–1600)  340 (820–2200)  65 (-400–1000) | 460 (230–560)  160 (59–700)  -180 (-330–330) | .83  .50  .44 | 150 (43–2600)  340 (140–1600)  90 (65–1900) | 460 (190–670)  190 (56–800)  -180 (-400–210) | .42  .64  .13 |
| TNFa | Acute samples  Convalescent samples  Difference | 39 (14–1200)  770 (7.6–1800)  650 (-510–2100) | 49 (25–1300)  240 (12–1500)  68 (-56–710) | .53  .65  .34 | 16 (13–4900)  1000 (720–2500)  1100 (650–3000) | 49 (27–1200)  160 (8.6–1300)  -3.9 (-290–840) | .32  .13  .08 |
| VEGF | Acute samples  Convalescent samples  Difference | 150 (35–210)  -  - | 40 (12–210)  -  - | .68  -  - | 130 (17–210)  -  - | 98 (15–210)  -  - | 1.0  -  - |
| Eotaxin-2 | Acute samples  Convalescent samples  Difference | 730 (210–960)  720 (360–940)  -52 (-400–320) | 910 (600–1100)  770 (520–1200)  -2.8 (-480–290) | .40  .65  .92 | 660 (340–890)  60 (370–830)  -52 (-91–47) | 780 (520–1000)  790 (450–1200)  -2.8 (-450–330) | .42  .45  1.0 |
| MCP-2 | Acute samples  Convalescent samples  Difference | 710 (100–900)  310(100–680)  -290 (-410– (-3.7)) | 930 (140–1300)  230 (95–550)  -770 (-2400– (-65)) | .32  .97  .15 | 840 (660–970)  320 (130–560)  -470 (-690– (-290)) | 690 (85–1300)  230 (72–640)  -300 (-820–14) | .72  .78  .61 |
| I-309 | Acute samples  Convalescent samples  Difference | 48 (26–97)  23 (12–43)  -14 (-49–7.5) | 21 (13–32)  24 (16–42)  -7.3 (-10–30) | **.049**  .88  .15 | 65 (36–100)  16 (11–42)  -15 (-46– (-14)) | 24 (16–36)  26 (17–41)  -7.3 (-17–11) | **.049**  .40  .10 |
| IL-16 | Acute samples  Convalescent samples  Difference | 78 (48–130)  44 (33–75)  -26 (-66–24) | 70 (49–81)  53 (38–77)  -7.3 (-10–30) | .52  .60  .46 | 79 (49–140)  34 (30–44)  -15 (-46– (-14)) | 70 (48–87)  54 (42–83)  -7.3 (-17–11) | .57  .07  .55 |
| TARC | Acute samples  Convalescent samples  Difference | 7.0 (4.1–26)  4.0 (2.2–12)  -0.56 (-13–6.5) | 3.0 (1.6–6.3)  4.1 (2.0–7.4)  -0.96 (-2.3–1.3) | **.03**  .65  1.0 | 5.9 (2.7–32)  2.6 (2.4–56)  0.56 (-2.2–33) | 4.0 (1.9–8.7)  5.2 (2.0–7.8)  -1.1 (-5.6–2.1) | .45  .85  .46 |
| Eotaxin-3 | Acute samples  Convalescent samples  Difference | 110 (110–170)  -  - | 110 (110–220)  -  - | .54  -  - | 110 (110–3400)  -  - | 110 (110–200)  -  - | .63  -  - |
| ENA-78 | Acute samples  Convalescent samples  Difference | 540 (110–1800)  260 (62–1700)  -99 (-1300–420) | 440 (170–2200)  130 (59–2300)  -200 (-380–49) | .62  .94  .92 | 640 (130–1300)  180 (36–1500)  -99 (-1100–800) | 440 (150–2200)  190 (69–2100)  -200 (-880–60) | .79  .64  .71 |

Acute sample, samples drawn at study entry; Convalescent sample, samples drawn at 2-week follow-up; Difference, difference in cytokine expression when comparing samples drawn at 2-week follow-up and at study entry.

Values are shown as medians (interquartile range).

Data were analyzed by Mann-Whitney U-test

**Supplementary Table 8**: Stimulants and their incubation times

| Priority | Stimulant | Stimulation time |
| --- | --- | --- |
| 1. | Anti-CD3/anti-CD28 | 24 h |
| 2. | Anti-CD3/anti-CD28 | 24 h |
| 3. | RV-16, 10 PFU/cell (5x10^6^PFU/well, 0,12 μg/well) | 5 d |
| 4. | Poly (I:C) (TLR3 agonist) | 24 h |
| 5. | RSV | 5 d |
| 6. | Ovomucoid (chicken egg) | 5 d |
| 7. | RV-16, 2.5 PFU/cell (1,25x10^6^PFU/well, 0,03 μg/well) | 5 d |
| 8. | Tetanus toxoid | 5 d |
| 9. | CL097 (TLR7/TLR8 agonist) | 24 h |
| 10. | Control | 5 d |
| 11. | Control | 24 h |
| 12. | RV-1B | 5 d |
| 13. | RV-14 | 5 d |

References for Supplementary Table 5

1. Tsou CL, Haskell CA, Charo IF. Tumor necrosis factor-alpha-converting enzyme mediates the inducible cleavage of fractalkine. *J Biol Chem*. 2001;276(48):44622-44626. doi:10.1074/jbc.M107327200

2. Cao J, Gan H, Xiao H, et al. Key protein-coding genes related to microglia in immune regulation and inflammatory response induced by epilepsy. *Math Biosci Eng MBE*. 2021;18(6):9563-9578. doi:10.3934/mbe.2021469

3. Zhang Y, Luxon BA, Casola A, Garofalo RP, Jamaluddin M, Brasier AR. Expression of respiratory syncytial virus-induced chemokine gene networks in lower airway epithelial cells revealed by cDNA microarrays. *J Virol*. 2001;75(19):9044-9058. doi:10.1128/JVI.75.19.9044-9058.2001

4. Zhivaki D, Lemoine S, Lim A, et al. Respiratory Syncytial Virus Infects Regulatory B Cells in Human Neonates via Chemokine Receptor CX3CR1 and Promotes Lung Disease Severity. *Immunity*. 2017;46(2):301-314. doi:10.1016/j.immuni.2017.01.010

5. Godwin MS, Jones M, Blackburn JP, et al. The chemokine CX3CL1/fractalkine regulates immunopathogenesis during fungal-associated allergic airway inflammation. *Am J Physiol Lung Cell Mol Physiol*. 2021;320(3):L393-L404. doi:10.1152/ajplung.00376.2020

6. Acosta-Rodriguez EV, Napolitani G, Lanzavecchia A, Sallusto F. Interleukins 1beta and 6 but not transforming growth factor-beta are essential for the differentiation of interleukin 17-producing human T helper cells. *Nat Immunol*. 2007;8(9):942-949. doi:10.1038/ni1496

7. Owczarczyk AB, Schaller MA, Reed M, Rasky AJ, Lombard DB, Lukacs NW. Sirtuin 1 Regulates Dendritic Cell Activation and Autophagy during Respiratory Syncytial Virus-Induced Immune Responses. *J Immunol Baltim Md 1950*. 2015;195(4):1637-1646. doi:10.4049/jimmunol.1500326

8. Nagarkar DR, Poposki JA, Comeau MR, et al. Airway epithelial cells activate TH2 cytokine production in mast cells through IL-1 and thymic stromal lymphopoietin. *J Allergy Clin Immunol*. 2012;130(1):225-232.e4. doi:10.1016/j.jaci.2012.04.019

9. Christiaansen AF, Syed MA, Ten Eyck PP, et al. Altered Treg and cytokine responses in RSV-infected infants. *Pediatr Res*. 2016;80(5):702-709. doi:10.1038/pr.2016.130

10. Mingyuan H, Kelley BJ, Charu R, et al. Inflammasome activation is required for human rhinovirus-induced airway inflammation in naive and allergen-sensitized mice. *Mucosal Immunol*. 2019;12(4):958-968. doi:http://dx.doi.org/10.1038/s41385-019-0172-2

11. Midulla F, Villani A, Panuska JR, et al. Respiratory syncytial virus lung infection in infants: immunoregulatory role of infected alveolar macrophages. *J Infect Dis*. 1993;168(6):1515-1519. doi:10.1093/infdis/168.6.1515

12. Han MW, Kim SH, Oh I, Kim YH, Lee J. Serum IL-1β can be a biomarker in children with severe persistent allergic rhinitis. *Allergy Asthma Clin Immunol Off J Can Soc Allergy Clin Immunol*. 2019;15:58. doi:10.1186/s13223-019-0368-8

13. Inhibition of uric acid or IL‐1β ameliorates respiratory syncytial virus immunopathology and development of asthma - Schuler - 2020 - Allergy - Wiley Online Library. Accessed November 25, 2021. https://onlinelibrary.wiley.com/doi/full/10.1111/all.14310

14. Elsner J, Petering H, Kluthe C, et al. Eotaxin-2 activates chemotaxis-related events and release of reactive oxygen species via pertussis toxin-sensitive G proteins in human eosinophils. *Eur J Immunol*. 1998;28(7):2152-2158. doi:10.1002/(SICI)1521-4141(199807)28:07<2152::AID-IMMU2152>3.0.CO;2-G

15. Forssmann U, Uguccioni M, Loetscher P, et al. Eotaxin-2, a Novel CC Chemokine that Is Selective for the Chemokine Receptor CCR3, and Acts Like Eotaxin on Human Eosinophil and Basophil Leukocytes. *J Exp Med*. 1997;185(12):2171-2176.

16. Bocchino V, Bertorelli G, Bertrand CP, et al. Eotaxin and CCR3 are up-regulated in exacerbations of chronic bronchitis. *Allergy*. 2002;57(1):17-22.

17. Lewis TC, Metitiri EE, Mentz GB, et al. Impact of community respiratory viral infections in urban children with asthma. *Ann Allergy Asthma Immunol Off Publ Am Coll Allergy Asthma Immunol*. 2019;122(2):175-183.e2. doi:10.1016/j.anai.2018.10.021

18. Anderson D, Jones AC, Gaido CM, et al. Differential Gene Expression of Lymphocytes Stimulated with Rhinovirus A and C in Children with Asthma. *Am J Respir Crit Care Med*. 2020;202(2):202-209. doi:10.1164/rccm.201908-1670OC

19. Sokulsky LA, Garcia-Netto K, Nguyen TH, et al. A Critical Role for the CXCL3/CXCL5/CXCR2 Neutrophilic Chemotactic Axis in the Regulation of Type 2 Responses in a Model of Rhinoviral-Induced Asthma Exacerbation. *J Immunol Baltim Md 1950*. 2020;205(9):2468-2478. doi:10.4049/jimmunol.1901350

20. Papadopoulos NG, Papi A, Meyer J, et al. Rhinovirus infection up-regulates eotaxin and eotaxin-2 expression in bronchial epithelial cells. *Clin Exp Allergy J Br Soc Allergy Clin Immunol*. 2001;31(7):1060-1066. doi:10.1046/j.1365-2222.2001.01112.x

21. Lewis TC, Metitiri EE, Mentz GB, et al. Influence of viral infection on the relationships between airway cytokines and lung function in asthmatic children. *Respir Res*. 2018;19(1):228. doi:10.1186/s12931-018-0922-9

22. Miller MD, Krangel MS. The human cytokine I-309 is a monocyte chemoattractant. *Proc Natl Acad Sci U S A*. 1992;89(7):2950-2954.

23. Liu J, Liu L, Kang W, et al. Cytokines/Chemokines: Potential Biomarkers for Non-paraneoplastic Anti-N-Methyl-D-Aspartate Receptor Encephalitis. *Front Neurol*. 2020;11:582296. doi:10.3389/fneur.2020.582296

24. Montes-Vizuet R, Vega-Miranda A, Valencia-Maqueda E, Negrete-García MC, Velásquez JR, Teran LM. CC chemokine ligand 1 is released into the airways of atopic asthmatics. *Eur Respir J*. 2006;28(1):59-67. doi:10.1183/09031936.06.00134304

25. Mutalithas K, Guillen C, Raport C, et al. Expression of CCR8 is increased in asthma. *Clin Exp Allergy*. 2010;40(8):1175-1185. doi:10.1111/j.1365-2222.2010.03504.x

26. Lai ST, Hung CH, Hua YM, Hsu SH, Jong YJ, Suen JL. T-helper 1-related chemokines in the exacerbation of childhood asthma. *Pediatr Int Off J Jpn Pediatr Soc*. 2008;50(1):99-102. doi:10.1111/j.1442-200X.2007.02533.x

27. Sallusto F, Lenig D, Mackay CR, Lanzavecchia A. Flexible programs of chemokine receptor expression on human polarized T helper 1 and 2 lymphocytes. *J Exp Med*. 1998;187(6):875-883. doi:10.1084/jem.187.6.875

28. Hansel TT, Tunstall T, Trujillo-Torralbo MB, et al. A Comprehensive Evaluation of Nasal and Bronchial Cytokines and Chemokines Following Experimental Rhinovirus Infection in Allergic Asthma: Increased Interferons (IFN-γ and IFN-λ) and Type 2 Inflammation (IL-5 and IL-13). *EBioMedicine*. 2017;19:128-138. doi:10.1016/j.ebiom.2017.03.033

29. M1-like macrophages are potent producers of anti-viral interferons and M1-associated marker-positive lung macrophages are decreased during rhinovirus-induced asthma exacerbations. Accessed November 25, 2021. https://www.ncbi.nlm.nih.gov/pmc/articles/PMC7152663/

30. Vojvoda V, Savić Mlakar A, Jergović M, et al. The increased type-1 and type-2 chemokine levels in children with acute RSV infection alter the development of adaptive immune responses. *BioMed Res Int*. 2014;2014:750521. doi:10.1155/2014/750521

31. Mehta AK, Croft M. Rhinovirus Infection Promotes Eosinophilic Airway Inflammation after Prior Exposure to House Dust Mite Allergen. *ImmunoHorizons*. 2020;4(8):498-507. doi:10.4049/immunohorizons.2000052

32. Monick MM, Powers LS, Hassan I, et al. Respiratory syncytial virus synergizes with Th2 cytokines to induce optimal levels of TARC/CCL17. *J Immunol Baltim Md 1950*. 2007;179(3):1648-1658. doi:10.4049/jimmunol.179.3.1648

33. Walz A, Schmutz P, Mueller C, Schnyder-Candrian S. Regulation and function of the CXC chemokine ENA-78 in monocytes and its role in disease. *J Leukoc Biol*. 1997;62(5):604-611. doi:10.1002/jlb.62.5.604

34. Rajan D, McCracken CE, Kopleman HB, et al. Human Rhinovirus Induced Cytokine/Chemokine Responses in Human Airway Epithelial and Immune Cells. *PLoS ONE*. 2014;9(12):e114322. doi:10.1371/journal.pone.0114322

35. Rajan D, Gaston KA, McCracken CE, Erdman DD, Anderson LJ. Response to rhinovirus infection by human airway epithelial cells and peripheral blood mononuclear cells in an in vitro two-chamber tissue culture system. *PloS One*. 2013;8(6):e66600. doi:10.1371/journal.pone.0066600

36. Donninger H, Glashoff R, Haitchi HM, et al. Rhinovirus induction of the CXC chemokine epithelial-neutrophil activating peptide-78 in bronchial epithelium. *J Infect Dis*. 2003;187(11):1809-1817. doi:10.1086/375246

37. Seckinger P, Lowenthal JW, Williamson K, Dayer JM, MacDonald HR. A urine inhibitor of interleukin 1 activity that blocks ligand binding. *J Immunol Baltim Md 1950*. 1987;139(5):1546-1549.

38. Yoon HJ, Zhu Z, Gwaltney JM, Elias JA. Rhinovirus regulation of IL-1 receptor antagonist in vivo and in vitro: a potential mechanism of symptom resolution. *J Immunol Baltim Md 1950*. 1999;162(12):7461-7469.

39. Oldford SA, Salsman SP, Portales-Cervantes L, et al. Interferon α2 and interferon γ induce the degranulation independent production of VEGF-A and IL-1 receptor antagonist and other mediators from human mast cells. *Immun Inflamm Dis*. 2018;6(1):176-189. doi:10.1002/iid3.211

40. Tabarani CM, Bonville CA, Suryadevara M, et al. Novel inflammatory markers, clinical risk factors and virus type associated with severe respiratory syncytial virus infection. *Pediatr Infect Dis J*. 2013;32(12):e437-442. doi:10.1097/INF.0b013e3182a14407

41. de Kluijver J, Grünberg K, Pons D, et al. Interleukin-1beta and interleukin-1ra levels in nasal lavages during experimental rhinovirus infection in asthmatic and non-asthmatic subjects. *Clin Exp Allergy J Br Soc Allergy Clin Immunol*. 2003;33(10):1415-1418. doi:10.1046/j.1365-2222.2003.01770.x

42. Godwin MS, Reeder KM, Garth JM, et al. IL-1RA regulates immunopathogenesis during fungal-associated allergic airway inflammation. *JCI Insight*. 2019;4(21):129055. doi:10.1172/jci.insight.129055

43. Akiki Z, Rava M, Diaz Gil O, et al. Serum cytokine profiles as predictors of asthma control in adults from the EGEA study. *Respir Med*. 2017;125:57-64. doi:10.1016/j.rmed.2017.03.002

44. Kimura A, Kishimoto T. IL-6: regulator of Treg/Th17 balance. *Eur J Immunol*. 2010;40(7):1830-1835. doi:10.1002/eji.201040391

45. Hurst SM, Wilkinson TS, McLoughlin RM, et al. Il-6 and its soluble receptor orchestrate a temporal switch in the pattern of leukocyte recruitment seen during acute inflammation. *Immunity*. 2001;14(6):705-714. doi:10.1016/s1074-7613(01)00151-0

46. Duhen T, Geiger R, Jarrossay D, Lanzavecchia A, Sallusto F. Production of interleukin 22 but not interleukin 17 by a subset of human skin-homing memory T cells. *Nat Immunol*. 2009;10(8):857-863. doi:10.1038/ni.1767

47. Spyridaki I, Taka S, Skevaki C, Trochoutsou A, Papadopoulos NG. In Vitro Effects of 5-Lipoxygenase Pathway Inhibition on Rhinovirus-Associated Bronchial Epithelial Inflammation. *Pulm Ther*. 2021;7(1):237-249. doi:10.1007/s41030-021-00152-x

48. Wang H, Bu L, Shu F, et al. Molecular Mechanism of Biofilm Locator Protein Kinase Dbf2p-related kinase 1 in Regulating Innate Immune Response to Interleukin 17-induced Viral Pneumonia. *Bioengineered*. Published online October 26, 2021. doi:10.1080/21655979.2021.1996316

49. Lui G, Wong CK, Chan M, et al. Host inflammatory response is the major marker of severe respiratory syncytial virus infection in older adults. *J Infect*. 2021;0(0). doi:10.1016/j.jinf.2021.09.024

50. Ramphul M, Welsh KG, May RD, et al. Sputum biomarkers during acute severe asthma attacks in children- a case-control study. *Acta Paediatr Oslo Nor 1992*. Published online November 13, 2021. doi:10.1111/apa.16186

51. Laan M, Prause O, Miyamoto M, et al. A role of GM-CSF in the accumulation of neutrophils in the airways caused by IL-17 and TNF-alpha. *Eur Respir J*. 2003;21(3):387-393. doi:10.1183/09031936.03.00303503

52. Griseri T, Arnold IC, Pearson C, et al. Granulocyte Macrophage Colony-Stimulating Factor-Activated Eosinophils Promote Interleukin-23 Driven Chronic Colitis. *Immunity*. 2015;43(1):187-199. doi:10.1016/j.immuni.2015.07.008

53. Rhinovirus-16 induced release of IP-10 and IL-8 is augmented by Th2 cytokines in a pediatric bronchial epithelial cell model - PubMed. Accessed November 25, 2021. https://pubmed.ncbi.nlm.nih.gov/24705919/

54. Subauste MC, Jacoby DB, Richards SM, Proud D. Infection of a human respiratory epithelial cell line with rhinovirus. Induction of cytokine release and modulation of susceptibility to infection by cytokine exposure. *J Clin Invest*. 1995;96(1):549-557. doi:10.1172/JCI118067

55. Abdullah SF. Granulocyte-Macrophage colony stimulating factor in asthmatic patients infected with respiratory syncytial virus. *Med J Malaysia*. 2021;76(2):177-182.

56. Nguyen-Thi-Dieu T, Le-Thi-Thu H, Duong-Quy S. The profile of leucocytes, CD3+, CD4+, and CD8+ T cells, and cytokine concentrations in peripheral blood of children with acute asthma exacerbation. *J Int Med Res*. 2017;45(6):1658-1669. doi:10.1177/0300060516680439

57. Nguyen-Thi-Dieu T, Le-Thi-Thu H, Le-Thi-Minh H, Pham-Nhat A, Duong-Quy S. Study of Clinical Characteristics and Cytokine Profiles of Asthmatic Children with Rhinovirus Infection during Acute Asthma Exacerbation at National Hospital of Pediatrics. *Can Respir J*. 2018;2018:9375967. doi:10.1155/2018/9375967

58. Kato M, Yamada Y, Maruyama K, Hayashi Y. Differential effects of corticosteroids on serum eosinophil cationic protein and cytokine production in rhinovirus- and respiratory syncytial virus-induced acute exacerbation of childhood asthma. *Int Arch Allergy Immunol*. 2011;155 Suppl 1:77-84. doi:10.1159/000327434

59. Naessens T, Schepens B, Smet M, et al. GM-CSF treatment prevents respiratory syncytial virus-induced pulmonary exacerbation responses in postallergic mice by stimulating alveolar macrophage maturation. *J Allergy Clin Immunol*. 2016;137(3):700-709.e9. doi:10.1016/j.jaci.2015.09.031

60. Ishioka T, Kimura H, Kita H, et al. Effects of respiratory syncytial virus infection and major basic protein derived from eosinophils in pulmonary alveolar epithelial cells (A549). *Cell Biol Int*. 2011;35(5):467-474. doi:10.1042/CBI20100255

61. Bradley LM, Dalton DK, Croft M. A direct role for IFN-gamma in regulation of Th1 cell development. *J Immunol Baltim Md 1950*. 1996;157(4):1350-1358.

62. Jazaeri S, Goldsmith AM, Jarman CR, Lee J, Hershenson MB, Lewis TC. Nasal interferon responses to community rhinovirus infections are similar in controls and children with asthma. *Ann Allergy Asthma Immunol Off Publ Am Coll Allergy Asthma Immunol*. 2021;126(6):690-695.e1. doi:10.1016/j.anai.2021.01.023

63. Ko YK, Zhang YL, Wee JH, Han DH, Kim HJ, Rhee CS. Human Rhinovirus Infection Enhances the Th2 Environment in Allergic and Non-allergic Patients with Chronic Rhinosinusitis. *Clin Exp Otorhinolaryngol*. 2021;14(2):217-224. doi:10.21053/ceo.2020.00444

64. van Erp EA, Lakerveld AJ, de Graaf E, et al. Natural killer cell activation by respiratory syncytial virus‐specific antibodies is decreased in infants with severe respiratory infections and correlates with Fc‐glycosylation. *Clin Transl Immunol*. 2020;9(2):e1112. doi:10.1002/cti2.1112

65. Li Z, Qu X, Liu X, et al. GBP5 Is an Interferon-Induced Inhibitor of Respiratory Syncytial Virus. *J Virol*. 2020;94(21):e01407-20. doi:10.1128/JVI.01407-20

66. Nickel R, Beck LA, Stellato C, Schleimer RP. Chemokines and allergic disease. *J Allergy Clin Immunol*. 1999;104(4 Pt 1):723-742. doi:10.1016/s0091-6749(99)70281-2

67. Williams TC, Jackson DJ, Maltby S, et al. Rhinovirus-induced CCL17 and CCL22 in Asthma Exacerbations and Differential Regulation by STAT6. *Am J Respir Cell Mol Biol*. 2021;64(3):344-356. doi:10.1165/rcmb.2020-0011OC

68. Olson MR, Hartwig SM, Varga SM. The number of respiratory syncytial virus (RSV)-specific memory CD8 T cells in the lung is critical for their ability to inhibit RSV vaccine-enhanced pulmonary eosinophilia. *J Immunol Baltim Md 1950*. 2008;181(11):7958-7968. doi:10.4049/jimmunol.181.11.7958

69. Roy B, Bhattacharjee A, Xu B, Ford D, Maizel AL, Cathcart MK. IL-13 signal transduction in human monocytes: phosphorylation of receptor components, association with Jaks, and phosphorylation/activation of Stats. *J Leukoc Biol*. 2002;72(3):580-589.

70. Minty A, Ferrara P, Caput D. Interleukin-13 effects on activated monocytes lead to novel cytokine secretion profiles intermediate between those induced by interleukin-10 and by interferon-gamma. *Eur Cytokine Netw*. 1997;8(2):189-201.

71. Rajput C, Han M, Ishikawa T, et al. Rhinovirus C Infection Induces Type 2 Innate Lymphoid Cell Expansion and Eosinophilic Airway Inflammation. *Front Immunol*. 2021;12:649520. doi:10.3389/fimmu.2021.649520

72. Shilovskiy IP, Yumashev KV, Nikolsky AA, Vishnyakova LI, Khaitov MR. Molecular and Cellular Mechanisms of Respiratory Syncytial Viral Infection: Using Murine Models to Understand Human Pathology. *Biochem Biokhimiia*. 2021;86(3):290-306. doi:10.1134/S0006297921030068

73. Andersson CK, Iwasaki J, Cook J, et al. Impaired airway epithelial cell wound-healing capacity is associated with airway remodelling following RSV infection in severe preschool wheeze. *Allergy*. 2020;75(12):3195-3207. doi:10.1111/all.14466

74. Zhang X, Zhang X, Zhang N, et al. Airway microbiome, host immune response and recurrent wheezing in infants with severe respiratory syncytial virus bronchiolitis. *Pediatr Allergy Immunol Off Publ Eur Soc Pediatr Allergy Immunol*. 2020;31(3):281-289. doi:10.1111/pai.13183

75. Fahey TJ, Tracey KJ, Tekamp-Olson P, et al. Macrophage inflammatory protein 1 modulates macrophage function. *J Immunol Baltim Md 1950*. 1992;148(9):2764-2769.

76. Kellar GG, Reeves SR, Barrow KA, Debley JS, Wight TN, Ziegler SF. Juvenile, but Not Adult, Mice Display Increased Myeloid Recruitment and Extracellular Matrix Remodeling during Respiratory Syncytial Virus Infection. *J Immunol Baltim Md 1950*. 2020;205(11):3050-3057. doi:10.4049/jimmunol.2000683

77. Blanco JCG, Richardson JY, Darnell MER, et al. Cytokine and chemokine gene expression after primary and secondary respiratory syncytial virus infection in cotton rats. *J Infect Dis*. 2002;185(12):1780-1785. doi:10.1086/340823

78. Al-Afif A, Alyazidi R, Oldford SA, et al. Respiratory syncytial virus infection of primary human mast cells induces the selective production of type I interferons, CXCL10, and CCL4. *J Allergy Clin Immunol*. 2015;136(5):1346-1354.e1. doi:10.1016/j.jaci.2015.01.042

79. Guerrero-Plata A, Kolli D, Hong C, Casola A, Garofalo RP. Subversion of pulmonary dendritic cell function by paramyxovirus infections. *J Immunol Baltim Md 1950*. 2009;182(5):3072-3083. doi:10.4049/jimmunol.0802262

80. Tripp RA, Moore D, Barskey A, et al. Peripheral blood mononuclear cells from infants hospitalized because of respiratory syncytial virus infection express T helper-1 and T helper-2 cytokines and CC chemokine messenger RNA. *J Infect Dis*. 2002;185(10):1388-1394. doi:10.1086/340505

81. Lewis TC, Henderson TA, Carpenter AR, et al. Nasal cytokine responses to natural colds in asthmatic children. *Clin Exp Allergy J Br Soc Allergy Clin Immunol*. 2012;42(12):1734-1744. doi:10.1111/cea.12005

82. Lee CG, Link H, Baluk P, et al. Vascular endothelial growth factor (VEGF) induces remodeling and enhances TH2-mediated sensitization and inflammation in the lung. *Nat Med*. 2004;10(10):1095-1103. doi:10.1038/nm1105

83. Moreno-Solís G, Torres-Borrego J, de la Torre-Aguilar MJ, Fernández-Gutiérrez F, Llorente-Cantarero FJ, Pérez-Navero JL. Analysis of the local and systemic inflammatory response in hospitalized infants with respiratory syncitial virus bronchiolitis. *Allergol Immunopathol (Madr)*. 2015;43(3):264-271. doi:10.1016/j.aller.2014.02.002

84. Pino M, Kelvin DJ, Bermejo-Martin JF, et al. Nasopharyngeal aspirate cytokine levels 1 yr after severe respiratory syncytial virus infection. *Pediatr Allergy Immunol Off Publ Eur Soc Pediatr Allergy Immunol*. 2009;20(8):791-795. doi:10.1111/j.1399-3038.2009.00868.x

85. Kuo C, Lim S, King NJC, et al. Rhinovirus infection induces expression of airway remodelling factors in vitro and in vivo. *Respirol Carlton Vic*. 2011;16(2):367-377. doi:10.1111/j.1440-1843.2010.01918.x

86. Leigh R, Oyelusi W, Wiehler S, et al. Human rhinovirus infection enhances airway epithelial cell production of growth factors involved in airway remodeling. *J Allergy Clin Immunol*. 2008;121(5):1238-1245.e4. doi:10.1016/j.jaci.2008.01.067

87. Hossny E, El-Awady H, Bakr S, Labib A. Vascular endothelial growth factor overexpression in induced sputum of children with bronchial asthma. *Pediatr Allergy Immunol Off Publ Eur Soc Pediatr Allergy Immunol*. 2009;20(1):89-96. doi:10.1111/j.1399-3038.2008.00730.x

88. Esposito S, Ierardi V, Daleno C, et al. Genetic polymorphisms and risk of recurrent wheezing in pediatric age. *BMC Pulm Med*. 2014;14:162. doi:10.1186/1471-2466-14-162

89. Loetscher P, Seitz M, Clark-Lewis I, Baggiolini M, Moser B. Monocyte chemotactic proteins MCP-1, MCP-2, and MCP-3 are major attractants for human CD4+ and CD8+ T lymphocytes. *FASEB J Off Publ Fed Am Soc Exp Biol*. 1994;8(13):1055-1060. doi:10.1096/fasebj.8.13.7926371

90. Karta MR, Gavala ML, Curran CS, et al. LPS modulates rhinovirus-induced chemokine secretion in monocytes and macrophages. *Am J Respir Cell Mol Biol*. 2014;51(1):125-134. doi:10.1165/rcmb.2013-0404OC
